# Raising AWaRe-ness of Antimicrobial Stewardship Challenges in Pediatric Emergency Care: Results from the PERFORM Study Assessing Consistency and Appropriateness of Antibiotic Prescribing Across Europe

**DOI:** 10.1093/cid/ciad615

**Published:** 2023-10-11

**Authors:** Laura Kolberg, Aakash Khanijau, Fabian J S van der Velden, Jethro Herberg, Tisham De, Rachel Galassini, Aubrey J Cunnington, Victoria J Wright, Priyen Shah, Myrsini Kaforou, Clare Wilson, Taco Kuijpers, Federico Martinón-Torres, Irene Rivero-Calle, Henriette Moll, Clementien Vermont, Marko Pokorn, Mojca Kolnik, Andrew J Pollard, Philipp K A Agyeman, Luregn J Schlapbach, Maria N Tsolia, Shunmay Yeung, Dace Zavadska, Werner Zenz, Nina A Schweintzger, Michiel van der Flier, Ronald de Groot, Effua Usuf, Marie Voice, Leonides Calvo-Bado, François Mallet, Katy Fidler, Michael Levin, Enitan D Carrol, Marieke Emonts, Ulrich von Both, The PERFORM Consortium, Michael Levin, Aubrey Cunnington, Tisham De, Jethro A Herberg, Myrsini Kaforou, Victoria J Wright, Lucas Baumard, Evangelos Bellos, Giselle D'Souza, Rachel Galassini, Dominic Habgood-Coote, Shea Hamilton, Clive Hoggart, Sara Hourmat, Heather Jackson, Naomi Lin, Ian Maconochie, Stephanie Menikou, Samuel Nichols, Ruud Nijman, Ivonne Pena Paz, Oliver Powell, Priyen Shah, Ortensia Vito, Clare Wilson, Molly Stevens, Eunjung Kim, Nayoung Kim, Amina Abdulla, Ladan Ali, Sarah Darnell, Rikke Jorgensen, Sobia Mustafa, Salina Persand, Katy Fidler, Julia Dudley, Vivien Richmond, Emma Tavliavini, Enitan D Carrol, Elizabeth Cocklin, Rebecca Jennings, Joanne Johnston, Aakash Khanijau, Simon Leigh, Nadia Lewis-Burke, Karen Newall, Sam Romaine, Andrew J Pollard, Rama Kandasamy, Stéphane Paulus, Michael J Carter, Daniel O'Connor, Sagida Bibi, Dominic F Kelly, Meeru Gurung, Stephen Thorson, Imran Ansari, David R Murdoch, Shrijana Shrestha, Zoe Oliver, Marieke Emonts, Emma Lim, Lucille Valentine, Karen Allen, Kathryn Bell, Adora Chan, Stephen Crulley, Kirsty Devine, Daniel Fabian, Sharon King, Paul McAlinden, Sam McDonald, Anne McDonnell, Ailsa Pickering, Evelyn Thomson, Amanda Wood, Diane Wallia, Phil Woodsford, Frances Baxter, Ashley Bell, Mathew Rhodes, Rachel Agbeko, Christine Mackerness, Bryan Baas, Lieke Kloosterhuis, Wilma Oosthoek, Tasnim Arif, Joshua Bennet, Kalvin Collings, Ilona van der Giessen, Alex Martin, Aqeela Rashid, Emily Rowlands, Gabriella de Vries, Fabian van der Velden, Joshua Soon, Lucille Valentine, Mike Martin, Ravi Mistry, Lucille Valentine, Shunmay Yeung, Juan Emmanuel Dewez, Martin Hibberd, David Bath, Alec Miners, Ruud Nijman, Elizabeth Fitchett, Colin Fink, Marie Voice, Leo Calvo-Bado, Federico Martinón-Torres, Antonio Salas, Fernando Álvez Gonz ález, Cristina Balo Farto, Ruth Barral-Arca, Marií Barreiro Castro, Xabier Bello, Mirian Ben García, Sandra Carnota, Miriam Cebey-López, María José Curras-Tuala, Carlos Dur án Su árez, Luisa García Vicente, Alberto Gómez-Carballa, Jose Gómez Rial, Pilar Lebor áns Iglesias, Federico Martinón-Torres, Nazareth Martinón-Torres, José María Martinón S ánchez, Belén Mosquera Pérez, Jacobo Pardo-Seco, Lidia Piñeiro Rodríguez, Sara Pischedda, Sara Rey V ázquez, Irene Rivero Calle, Carmen Rodríguez-Tenreiro, Lorenzo Redondo-Collazo, Miguel Sadiki Ora, Antonio Salas, Sonia Serén Fern ández, Cristina Serén Trasorras, Marisol Vilas Iglesias, Henriëtte A Moll, Clementien L Vermont, Dorine M Borensztajn, Nienke N Hagedoorn, Chantal Tan, Joany Zachariasse, W Dik, Ronald de Groot, Michiel van der Flier, Marien I de Jonge, Koen van Aerde, Wynand Alkema, Bryan van den Broek, Jolein Gloerich, Alain J van Gool, Stefanie Henriet, Martijn Huijnen, Ria Philipsen, Esther Willems, G P J M Gerrits, M van Leur, J Heidema, L de Haan, C J Miedema, C Neeleman, C C Obihara, G A Tramper-Stranders, Taco Kuijpers, Ilse Jongerius, J M van den Berg, D Schonenberg, A M Barendregt, D Pajkrt, M van der Kuip, A M van Furth, Evelien Sprenkeler, Judith Zandstra, G van Mierlo, J Geissler, Dace Zavadska, Anda Balode, Arta Bārzdiņa, Dārta Deksne, Dace Gardovska, Dagne Grāvele, Ilze Grope, Anija Meiere, Ieva Nokalna, Jana Pavāre, Zanda Pučuka, Katrīna Selecka, Aleksandra Rudzāte, Dace Svile, Urzula Nora Urbāne, Werner Zenz, Benno Kohlmaier, Nina A Schweintzger, Manfred G Sagmeister, Daniela S Kohlfürst, Christoph Zurl, Alexander Binder, Susanne Høsele, Manuel Leitner, Lena Pølz, Glorija Rajic, Sebastian Bauchinger, Hinrich Baumgart, Martin Benesch, Astrid Ceolotto, Ernst Eber, Siegfried Gallistl, Gunther Gores, Harald Haidl, Almuthe Hauer, Christa Hude, Markus Keldorfer, Larissa Krenn, Heidemarie Pilch, Andreas Pfleger, Klaus Pfurtscheller, Gudrun Nordberg, Tobias Niedrist, Siegfried Rødl, Andrea Skrabl-Baumgartner, Matthias Sperl, Laura Stampfer, Volker Strenger, Holger Till, Andreas Trobisch, Sabine Løffler, Ulrich von Both, Laura Kolberg, Manuela Zwerenz, Judith Buschbeck, Christoph Bidlingmaier, Vera Binder, Katharina Danhauser, Nikolaus Haas, Matthias Griese, Tobias Feuchtinger, Julia Keil, Matthias Kappler, Eberhard Lurz, Georg Muench, Karl Reiter, Carola Schoen, Maria Tsolia, Irini Eleftheriou, Maria Tambouratzi, Antonis Marmarinos, Marietta Xagorari, Kelly Syggelou, Philipp Agyeman, Luregn J Schlapbach, Christoph Aebi, Verena Wyss, Mariama Usman, Eric Giannoni, Martin Stocker, Klara M Posfay-Barbe, Ulrich Heininger, Sara Bernhard-Stirnemann, Anita Niederer-Loher, Christian Kahlert, Giancarlo Natalucci, Christa Relly, Thomas Riedel, Christoph Aebi, Christoph Berger, Marko Pokorn, Mojca Kolnik, Katarina Vincek, Tina Plankar Srovin, Natalija Bahovec, Petra Prunk, Veronika Osterman, Tanja Avramoska, François Mallet, Karen Brengel-Pesce, Alexandre Pachot, Marine Mommert, Effua Usuf, Kalifa Bojang, Syed M A Zaman, Fatou Secka, Suzanne Anderson, Anna RocaIsatou Sarr, Momodou Saidykhan, Saffiatou Darboe, Samba Ceesay, Umberto D'alessandro, Ching-Fen Shen, Ching-Chuan Liu, Shih-Min Wang

**Affiliations:** Dr. von Hauner Children's Hospital, Division Pediatric Infectious Diseases, University Hospital, LMU Munich, Munich, Germany; Department of Infectious Diseases, Alder Hey Children's Hospital, Liverpool, United Kingdom; Institute of Infection, Veterinary and Ecological Sciences, University of Liverpool, Liverpool, United Kingdom; Pediatric Immunology, Infectious Diseases & Allergy Department, Great North Children's Hospital, Newcastle Upon Tyne, United Kingdom; Translational and Clinical Research Institute, Newcastle University, Newcastle Upon Tyne, United Kingdom; Section of Pediatric Infectious Disease, Department of Infectious Disease, Faculty of Medicine, Imperial College London, London, United Kingdom; Section of Pediatric Infectious Disease, Department of Infectious Disease, Faculty of Medicine, Imperial College London, London, United Kingdom; Section of Pediatric Infectious Disease, Department of Infectious Disease, Faculty of Medicine, Imperial College London, London, United Kingdom; Section of Pediatric Infectious Disease, Department of Infectious Disease, Faculty of Medicine, Imperial College London, London, United Kingdom; Section of Pediatric Infectious Disease, Department of Infectious Disease, Faculty of Medicine, Imperial College London, London, United Kingdom; Section of Pediatric Infectious Disease, Department of Infectious Disease, Faculty of Medicine, Imperial College London, London, United Kingdom; Section of Pediatric Infectious Disease, Department of Infectious Disease, Faculty of Medicine, Imperial College London, London, United Kingdom; Section of Pediatric Infectious Disease, Department of Infectious Disease, Faculty of Medicine, Imperial College London, London, United Kingdom; Amsterdam University Medical Center, Location Academic Medical Center, Department of Pediatric Immunology, Rheumatology and Infectious Diseases, University of Amsterdam, Amsterdam, The Netherlands; Translational Pediatrics and Infectious Diseases, Hospital Clinico Universitario de Santiago de Compostela, Santiago De Compostela, Spain; Translational Pediatrics and Infectious Diseases, Hospital Clinico Universitario de Santiago de Compostela, Santiago De Compostela, Spain; Department of General Pediatrics, Erasmus MC-Sophia Children's Hospital, Rotterdam, The Netherlands; Department of General Pediatrics, Erasmus MC-Sophia Children's Hospital, Rotterdam, The Netherlands; Department of Pediatrics, Division of Pediatric Infectious Diseases & Immunology, Erasmus MC-Sophia Children's Hospital, Rotterdam, The Netherlands; Univerzitetni Klinični Center, Department of Infectious Diseases, University Medical Centre Ljubljana, Ljubljana, Slovenia; University Medical Center Ljubljana, University Children's Hospital, Ljubljana, Slovenia; Oxford Vaccine Group, Department of Pediatrics, University of Oxford, Oxford, United Kingdom; NIHR Oxford Biomedical Research Centre, Oxford University Hospitals Trust, Oxford, United Kingdom; Department of Pediatrics, Inselspital, Bern University Hospital, University of Bern, Bern, Switzerland; Department of Intensive Care and Neonatology, and Children's Research Center, University Children's Hospital Zurich, Zurich, Switzerland; Second Department of Pediatrics, Children's Hospital ‘P. and A. Kyriakou,’ National and Kapodistrian University of Athens, Athens, Greece; Clinical Research Department, London School of Hygiene and Tropical Medicine, London, United Kingdom; Children Clinical University Hospital, Department of Pediatrics, Rīgas Stradina Universitāte, Riga, Latvia; Department of Pediatrics and Adolescent Medicine, Division of General Pediatrics, Medical University of Graz, Graz, Austria; Department of Pediatrics and Adolescent Medicine, Division of General Pediatrics, Medical University of Graz, Graz, Austria; Pediatric Infectious Diseases and Immunology, Amalia Children's Hospital, Radboud University Medical Center, Nijmegen, The Netherlands; Wilhelmina Children's Hospital, Pediatric Infectious Diseases and Immunology, University Medical Center Utrecht, Utrecht, The Netherlands; Pediatric Infectious Diseases and Immunology, Amalia Children's Hospital, Radboud University Medical Center, Nijmegen, The Netherlands; Medical Research Council Unit, The Gambia at London School of Hygiene and Tropical Medicine, Fajara, The Gambia; Micropathology Ltd, The Venture Center, University of Warwick Science Park, Coventry, United Kingdom; Micropathology Ltd, The Venture Center, University of Warwick Science Park, Coventry, United Kingdom; Joint Research Unit Hospice Civils de Lyon–bioMérieux, Centre Hospitalier Lyon Sud, Pierre-Bénite, France; Academic Department of Pediatrics, Royal Alexandra Children's Hospital, Brighton, United Kingdom; Brighton and Sussex Medical School, University of Sussex, East Sussex, United Kingdom; Section of Pediatric Infectious Disease, Department of Infectious Disease, Faculty of Medicine, Imperial College London, London, United Kingdom; Department of Infectious Diseases, Alder Hey Children's Hospital, Liverpool, United Kingdom; Institute of Infection, Veterinary and Ecological Sciences, University of Liverpool, Liverpool, United Kingdom; Pediatric Immunology, Infectious Diseases & Allergy Department, Great North Children's Hospital, Newcastle Upon Tyne, United Kingdom; Translational and Clinical Research Institute, Newcastle University, Newcastle Upon Tyne, United Kingdom; NIHR Newcastle Biomedical Research Centre, Newcastle Upon Tyne Hospitals NHS Trust and Newcastle University, Newcastle Upon Tyne, United Kingdom; Dr. von Hauner Children's Hospital, Division Pediatric Infectious Diseases, University Hospital, LMU Munich, Munich, Germany; German Center for Infection Research (DZIF), Partner Site Munich, Munich, Germany

**Keywords:** antimicrobial stewardship, pediatric emergency care, antibiotic prescription, AWaRe, infectious diseases

## Abstract

**Background:**

Optimization of antimicrobial stewardship is key to tackling antimicrobial resistance, which is exacerbated by overprescription of antibiotics in pediatric emergency departments (EDs). We described patterns of empiric antibiotic use in European EDs and characterized appropriateness and consistency of prescribing.

**Methods:**

Between August 2016 and December 2019, febrile children attending EDs in 9 European countries with suspected infection were recruited into the PERFORM (Personalised Risk Assessment in Febrile Illness to Optimise Real-Life Management) study. Empiric systemic antibiotic use was determined in view of assigned final “bacterial” or “viral” phenotype. Antibiotics were classified according to the World Health Organization (WHO) AWaRe classification.

**Results:**

Of 2130 febrile episodes (excluding children with nonbacterial/nonviral phenotypes), 1549 (72.7%) were assigned a bacterial and 581 (27.3%) a viral phenotype. A total of 1318 of 1549 episodes (85.1%) with a bacterial and 269 of 581 (46.3%) with a viral phenotype received empiric systemic antibiotics (in the first 2 days of admission). Of those, the majority (87.8% in the bacterial and 87.0% in the viral group) received parenteral antibiotics. The top 3 antibiotics prescribed were third-generation cephalosporins, penicillins, and penicillin/β-lactamase inhibitor combinations. Of those treated with empiric systemic antibiotics in the viral group, 216 of 269 (80.3%) received ≥1 antibiotic in the “Watch” category.

**Conclusions:**

Differentiating bacterial from viral etiology in febrile illness on initial ED presentation remains challenging, resulting in a substantial overprescription of antibiotics. A significant proportion of patients with a viral phenotype received systemic antibiotics, predominantly classified as WHO Watch. Rapid and accurate point-of-care tests in the ED differentiating between bacterial and viral etiology could significantly improve antimicrobial stewardship.

## Teaser

Differentiating bacterial or viral etiology of febrile illness on initial presentation is challenging. Suspected bacterial etiology paired with clinical uncertainty results in high use of antibiotics in the World Health Organization “Watch” category, particularly in patients with severe viral disease.

Febrile illness is among the most common pediatric presentations at the emergency department (ED), contributing to 14% of attendances [1]. Most febrile children attending EDs likely have a self-limiting or viral infection, with the incidence of serious bacterial infection ranging from 5%–15% [2, 3], but approximately 33% receive antibiotics, and frequently broad-spectrum antibiotics [3, 4]. Discrepancy between confirmed bacterial infection and antibiotic prescription is partly explained by diagnostic uncertainty; in up to a fifth of presentations, no obvious cause of fever is found on clinical examination [5, 6]. This uncertainty gives rise to antimicrobial use for nonbacterial infections and drives antimicrobial resistance (AMR).

Given the ever-increasing threat to public health posed by AMR [7], judicious use of antimicrobials in the pediatric emergency setting is vital. The World Health Organization (WHO) global action plan encourages identifying patterns of antimicrobial use to optimize antimicrobial stewardship (AMS) programs in pediatric settings [8].

Work in recent years has shown that AMS programs need to be improved in pediatric primary, secondary, and tertiary care [3, 9, 10]. While there are significant data on prescribing patterns in primary care and the inpatient setting, there are fewer data on antimicrobial use in EDs [11–13].

The WHO AWaRe classification, developed as a tool to optimize antimicrobial use [14] classifies antibiotics into 3 AMS categories: Access, narrow-spectrum antibiotics considered as first- or second-line options for common infections; Watch, key targets for AMS initiatives, with higher potential for inducing resistance, and Reserve, “last-resort” options against multidrug-resistant or extensively drug-resistant bacteria [15].

We aimed to describe patterns of empiric systemic antibiotic use in the context of the WHO AWaRe classification to assess how the use of Access, Watch, and Reserve antibiotics varies across European pediatric EDs, microbiological etiology and clinical syndromes. We evaluated the appropriateness and consistency of antibiotic prescribing.

## METHODS

### Study Population and Study Design

The study population consisted of children (aged 0–18 years) enrolled in the Personalised Risk Assessment in Febrile Illness to Optimise Real-Life Management (PERFORM) study between August 2016 and December 2019. PERFORM is a multicenter, prospective, observational cohort study seeking to improve the diagnosis of febrile illness in children across Europe (https://www.perform2020.org/). Children who attended EDs with suspicion of infection and were considered to require blood tests were recruited, independent of the decision for inpatient or outpatient care [16]. Clinical data were prospectively collected by local study teams. Each patient was assigned final syndrome classification(s) and a phenotype by local study teams, including local principal investigators, based on collected clinical and laboratory data, following clear guidance of the PERFORM phenotyping algorithm (Supplementary Figure 1) [17]. To ensure accuracy and consistency of data entry and phenotyping, regular cross-site checks of randomly selected patients were performed. This was complemented by electronic quality control for all patients in the database.

Written informed consent was obtained from legal guardians of participants or participants themselves, per national guidance. The study was approved by the ethics committees of local recruitment sites and the coordinating site (Imperial College London; 16/LO/1684) (Supplementary Table 1).

### Recording of Diagnoses and Clinical Syndrome Classifications

Initial and final diagnoses were recorded from prespecified lists of clinical syndrome classifications within the case record form (CRF), by the patients’ clinicians (Supplementary Table 2). Presumed etiology was recorded with initial diagnosis and was categorized as "presumed bacterial,” “presumed viral,” “presumed noninfectious” (eg, for inflammatory syndromes), or unspecified.

### Phenotyping of Participants

**Figure 1.**
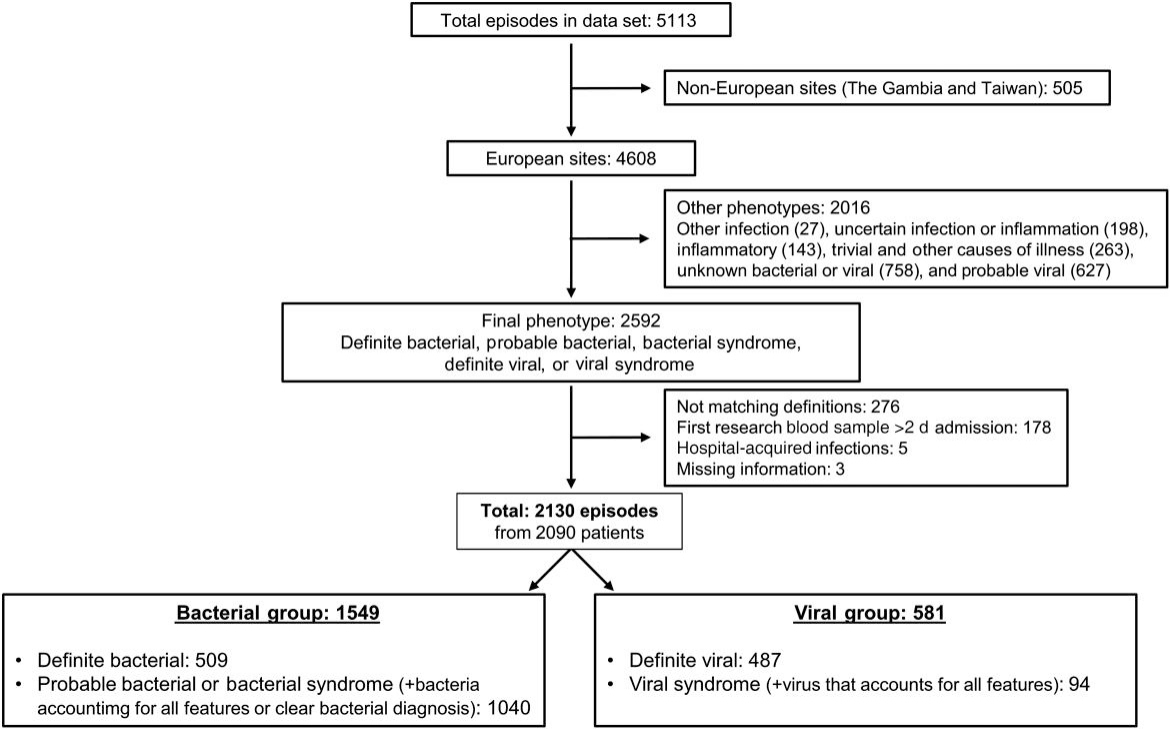
Febrile episodes selected for analysis.

Febrile episodes were phenotyped using the PERFORM phenotyping algorithm (Supplementary Figure 1) and then analyzed in 1 of 2 groups defined as “bacterial” or “viral” [17]. For the bacterial group, we included patients with a “definite bacterial” phenotype (509 episodes), and those with a “probable bacterial” (599 episodes) or “bacterial syndrome” (441 episodes) phenotype (with bacteria detected accounting for all features or clear bacterial diagnosis). Patients who were assigned a final “definite viral” (487 episodes) or “viral syndrome” (with virus detected accounting for all features) (94 episodes) phenotype were included in the viral group. Patients categorized as “probable viral” were not included, because no definitive causative viral pathogen had been identified. Participants with hospital-acquired infections (symptom/fever onset >2 days after presentation to hospital) were excluded from the analysis, as well as participants with unknown symptom and fever onset and those for whom research blood samples could not be obtained within 2 days after admission (Figure 1).

### Antibiotic Classes and AWaRe Classification

Empiric systemic antibiotics were defined as those prescribed within 2 days after presentation to hospital. These were categorized by antibiotic classes following the 3 WHO AWaRe categories (Access, Watch, and Reserve) (Supplementary Table 3).

### Outcomes

Primary outcomes were appropriateness and consistency of empiric antibiotic use, considering the final phenotype and syndrome classification (Supplementary Table 4). For the bacterial group, withholding antibiotics was defined as inappropriate, unless in certain diagnoses (Supplementary Table 5). This judgment was made by review of final syndrome classification by study clinicians. For the viral group, any antibiotic use was defined as inappropriate (Supplementary Table 4). In addition, for the bacterial group, we described antibiotic use, stratified by both initial and final syndrome classification. Only patients with a single main syndrome classification (Supplementary Table 2) were included in the latter analysis, to remove conflicting indications for antibiotic use. We evaluated consistency considering the recorded presumed etiology (bacterial vs viral or noninfectious), where consistency was defined as using antibiotics only when the presumed etiology was bacterial. A secondary outcome was describing empiric antibiotic use for the 3 most common bacterial and viral pathogens.

### Statistical Analysis

Distribution of variables was described in absolute numbers and percentages. We used χ^2^ tests to determine whether the variables explored were independent of each other, using R software, version 4.0.2 (R Foundation for Statistical Computing) [18].

## RESULTS

We included 2130 febrile episodes (from 2090 patients) from 9 European countries in this study. Of these episodes, 1549 (72.7%) were classified as bacterial, and 581 (27.3%) as viral. Of the 2130 episodes, 1156 (54.3%) were in male participants. Their median age was 5 years (bacterial) and 3 years (viral). Most patients (714 episodes, 33.5%) were from UK sites (Table 1). The most common main initial and final syndrome classifications were lower respiratory tract infection (initial, 421 [19.8%]; final, 501 [23.5%]) and upper respiratory tract infection (URTI) (initial. 399 [18.7%]; final, 435 [20.0%]) (Supplementary Table 6).

**Table 1.** Patient Characteristics for Febrile Episodes Included in Analysis (n = 2130)

Characteristic	Episodes, No. (%)			*P* Value^a^
	Bacterial (n = 1549)	Viral (n = 581)	Total (n = 2130)	
Sex				.57
Male	847 (54.7)	309 (53.2)	1156 (54.3)	
Female	702 (45.3)	272 (46.8)	974 (45.7)	
Age, y				<.001
<1	220 (14.2)	160 (27.5)	380 (17.8)	
1–5	640 (41.3)	240 (41.3)	880 (41.3)	
6–14	553 (35.7)	150 (25.8)	703 (33.0)	
15–17	136 (8.8)	31 (5.3)	167 (7.8)	
Country				<.001
Austria	148 (9.6)	46 (7.9)	194 (9.1)	
Germany	21 (1.4)	10 (1.7)	31 (1.5)	
Greece	149 (9.6)	107 (18.4)	256 (12.0)	
Latvia	194 (12.5)	46 (7.9)	240 (11.3)	
Netherlands	186 (12.0)	55 (9.5)	241 (11.3)	
Slovenia	127 (8.2)	24 (4.1)	151 (7.1)	
Spain	152 (9.8)	64 (11.0)	216 (10.1)	
Switzerland	79 (5.1)	8 (1.4)	87 (4.1)	
United Kingdom	493 (31.8)	221 (38.0)	714 (33.5)	
Regional Ancestry ^b^				<.001
European	1316 (85.0)	447 (77.0	1763 (82.8)	
(North) African	35 (2.3)	22 (3.8)	57 (2.7)	
Asian	58 (3.7)	49 (8.4)	107 (5.0)	
Middle Eastern	36 (2.3)	26 (4.5)	62 (2.9)	
South American	3 (0.2)	0 (0.0)	3 (0.1)	
Other	10 (0.6)	7 (1.2)	17 (0.4)	
Mixed	26 (1.7)	14 (2.4)	40 (1.9)	
Antibiotic use within 7 d before presentation				.13
Yes	370 (23.9)	120 (20.7)	490 (23.0)	
No	1179 (76.1)	461 (79.3)	1640 (77.0)	
Patient status after presentation to ED				.36
Admitted	1305 (84.2)	477 (82.1)	1782 (83.7)	
Discharged	210 (13.6)	86 (14.8)	296 (13.9)	
Transferred	30 (1.9)	14 (2.4)	44 (2.1)	
Unknown	4 (0.3)	4 (0.7)	8 (0.4)	

Abbreviation: ED, emergency department.

^a^ *P* values calculated using χ^2^ test.

^b^Regional Ancestry was missing or unknown in 81 episodes (3.8%).

Overall, in 1587 episodes (74.5%) patients received empiric systemic antibiotics, with significant variation between countries. The 3 most frequently prescribed antibiotics in both groups (bactrial and viral) were third-generation cephalosporins (prescribed in 34.6% vs 60.6%, respectively, of those who received antibiotics), penicillin/β-lactamase inhibitor combinations (31.1% and 24.5%) and penicillins (26.9% and 23.4%) (Supplementary Tables 7 and 8).

### Appropriateness of Antibiotic Use

Of 1549 patients presenting with a febrile episode in the bacterial group, 1318 (85.1%) received empiric systemic antibiotics administered parenterally (intravenously or intramuscularly) in 1157 of 1318 (87.8%). In the bacterial group, 231 patients presenting with a febrile episode (14.9%) did not receive empiric antibiotics; in 120 (7.7%), withholding antibiotics was considered inappropriate (Supplementary Table 5). Of 581 (46.3%) patients presenting with a febrile episode in the viral group, 269 (46.3%) received inappropriate empiric antibiotics (87.0% intravenous or intramuscular).

**Figure 2.**
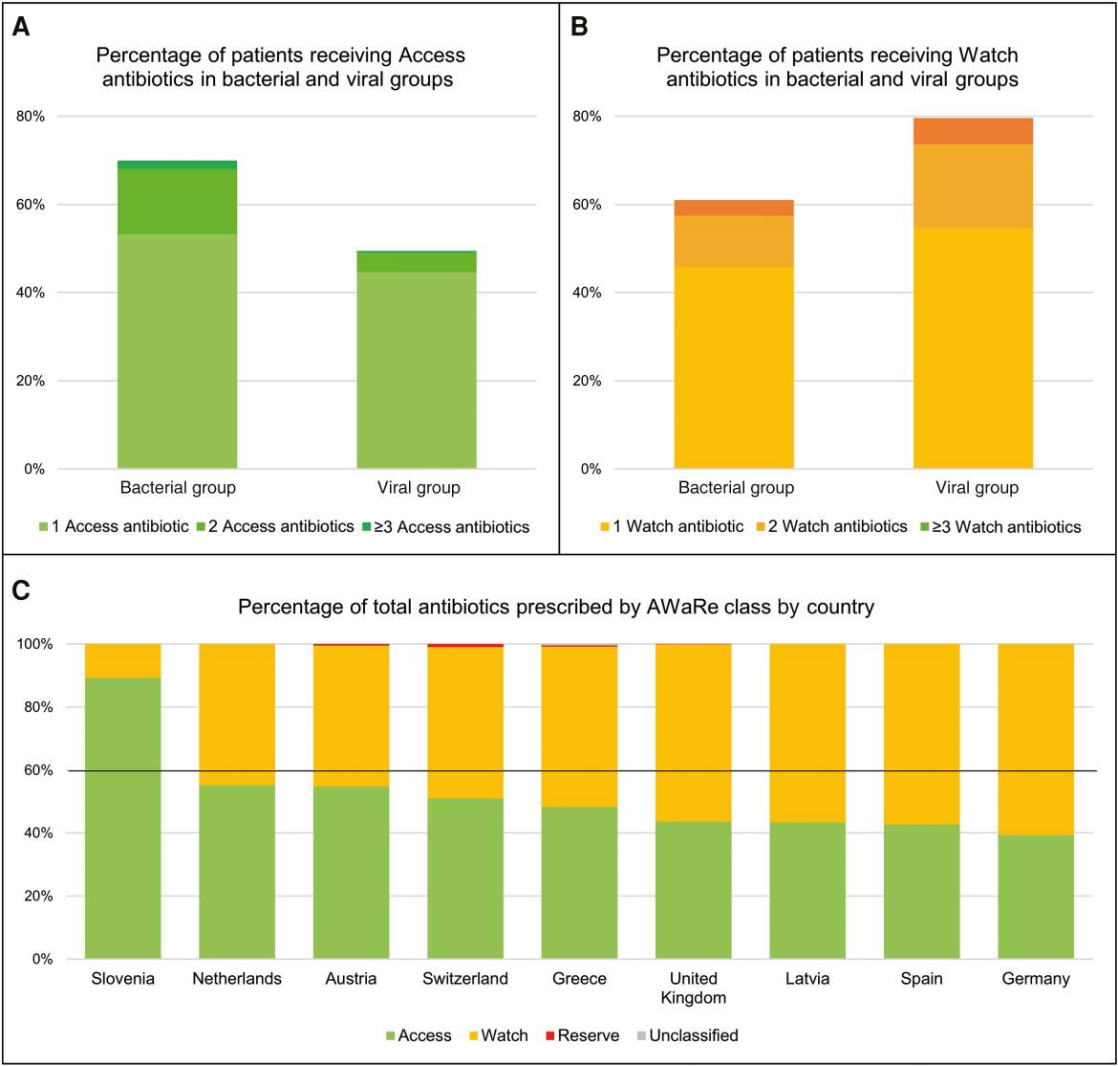
Proportions of Access, Watch, and Reserve antibiotics, in the World Health Organization (WHO) AWaRe classification, prescribed in the “bacterial” and “viral” groups. Line in (*C*) indicates the WHO target for 60% Access use.

Of patients receiving antibiotics for a febrile episode in the bacterial group, 70.0% received ≥1 Access antibiotic and 61.0% ≥ 1 Watch antibiotic. Of patients receiving antibioticsfor a febrile episode in the viral group, 50.2% received ≥1 Access antibiotic and 80.3% ≥1 Watch antibiotic (Figure 2*A* and 2*B* and Supplementary Tables 7 and 8). There was significant variation in the proportions of AWaRe antibiotics used in different countries, with Slovenia having the highest (89.2%) and Germany the lowest (39.3%) proportion of Access antibiotic use. We identified 49.1% Access use across all countries. (Figure 2*C*).

**Figure 3.**
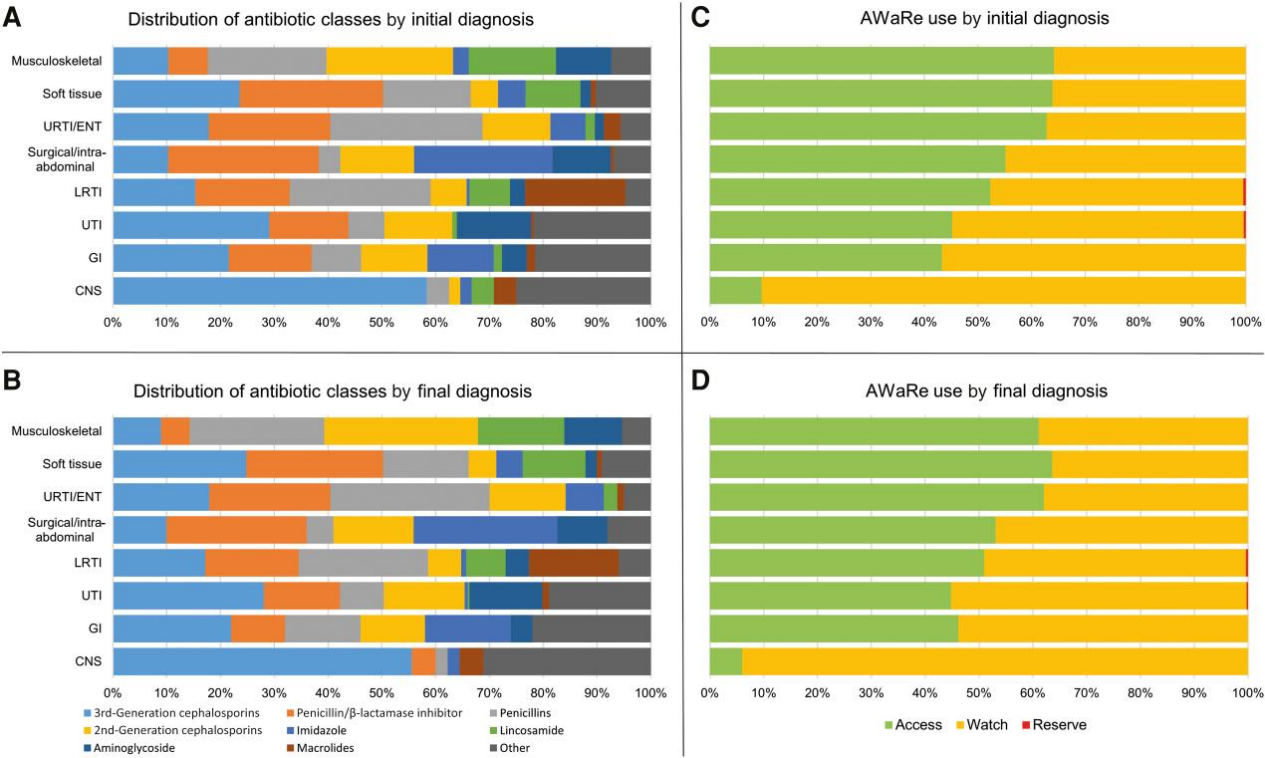
Distribution of antibiotics (classes and World Health Organization AWaRe classification) by single main initial and final syndrome classification in the “bacterial” group. The “other” category includes first-generation cephalosporins, glycopeptide, fluoroquinolones, carbapenems, dihydrofolate reductase inhibitors, fourth-generation cephalosporins, nitrofurantoin, oxazolidinones, rifamycins, tetracyclines, amphenicols and unknown antibiotics. Abbreviations: CNS, central nervous system; GI, gastrointestinal; LRTI, lower respiratory tract infection; URTI/ENT, upper respiratory tract infection or ear, nose, and throat; UTI, urinary tract infection.

Most patients with a single initial main syndrome classification—1326 of 1520 febrile episodes (87.2%)—were attributed the same main final syndrome classification (Supplementary Figure 2). Among patients in the bacterial group with a single initial syndrome classification, the most common antibiotic classes prescribed varied by syndrome—however, penicillins, penicillin/β-lactamase inhibitor combinations, and second- and third-generation cephalosporins accounted for the majority of antibiotics (Figure 3*A* and 3*C*). The central nervous system showed the highest proportion of Watch antibiotic use. In patients with a single final syndrome classification, antibiotic choice and the use of Watch antibiotics followed a similar pattern (Figure 3*B* and 3*D*).

### Consistency of Antibiotic Use

**Figure 4.**
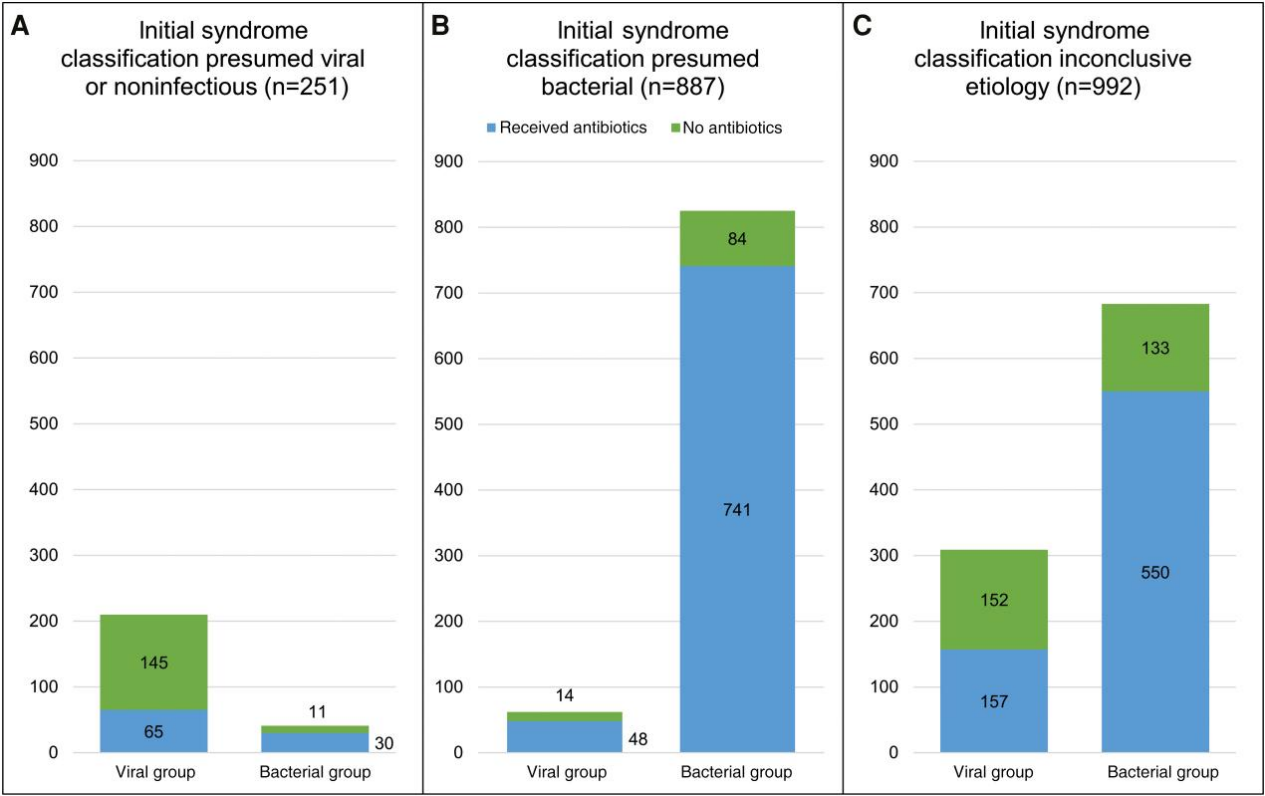
Number of febrile episodes with “bacterial” or “viral” phenotype receiving antibiotics in relation to the presumed etiology of the initial syndrome classification.

Of 251 episodes with a presumed viral or noninfectious etiology, 41 (16.3%) were subsequently phenotyped as bacterial, of which 30 (73.2%) received antibiotics; the remaining 210 episodes (83.7%) were assigned a viral phenotype, of which 65 (31.0%) received antibiotics (Figure 4*A*). Of the 251 episodes in this group, 95 (37.8%) received antibiotics inconsistent with the presumed etiology. An age-stratified overview of antibiotic prescribing patterns for patients with an initial viral or noninfectious initial syndrome classification is shown in Supplementary Table 9.

Of 887 episodes with a presumed bacterial etiology, 825 (93.0%) were assigned a final bacterial phenotype, of which 741 (89.8%) received antibiotics. Of 62 episodes (7.0%) assigned a final viral phenotype, 48 (77.4%) received antibiotics (Figure 4*B*). Of the 887 episodes in this group, 98 (11.0%) did not receive antibiotics, which is inconsistent with the presumed etiology.

For episodes in which the initial syndrome classification included both presumed bacterial and viral etiologies, unspecified infection, or undifferentiated fever (n = 992), 683 (68.9%) were attributed a final bacterial phenotype of which 550 (80.5%) received antibiotics. Of 992 episodes, 309 (31.1%) were attributed a final viral phenotype, of which 157 (50.8%) received antibiotics (Figure 4*C*).

The most common pathogens in the bacterial group were *Escherichia coli, Streptococcus pyogenes* (group A *Streptococcus*), and *Staphylococcus aureus* (Supplementary Table 10). Many patients with infections caused by these 3 pathogens received systemic Watch antibiotics (63.3%, 47.8%, and 49.0% respectively) (Supplementary Table 11). The most common viral pathogens in the viral group were influenza A/B, rhino/enterovirus, and respiratory syncytial virus (RSV) (Supplementary Table 10). Among patients with these pathogens, many received antibiotics (35.3%, 64.0%, and 66.7%. respectively). Of all the patients who received systemic antibiotics, 79.7% received ≥1 Watch antibiotic (73.8% with influenza A and B, 84.2% with rhino/enterovirus, and 81.0% with RSV) (Supplementary Table 12).

## DISCUSSION

We assessed the appropriateness and consistency of empiric antibiotic use in European EDs using data from the PERFORM study, for children attending EDs with suspected infection and considered to require blood tests, and we describe antibiotic use per the AWaRe classifications.

We demonstrated that a significant proportion of children within this cohort receive systemic antibiotics, including substantial use of Watch antibiotics, with some variation between European countries. Across the cohort, the proportion of empiric antibiotics prescribed from the Access category (49.1%) fell below the WHO target of 60%, illustrating an excessive use of Watch antibiotics [14]. A national AWaRe-based analysis of prescription data from pediatric outpatient and EDs in 16 secondary and tertiary care hospitals in China reported similar results. Watch antibiotics were most frequently prescribed (82.2%), third-generation cephalosporins (43.3%) in particular [19]. Variation in antibiotic use is not limited to EDs, and continuous monitoring of Watch antibiotic use in pediatric hospitals will be important for AMS interventions.

We show that many patients with viral illness receive empiric antibiotics at presentation to the ED. Of particular note, the proportion of patients receiving Watch antibiotics was higher in the viral than in the bacterial group (Figure 2).

In a small proportion (7.7%) of febrile episodes from patients with a bacterial phenotype, empiric antibiotics were withheld, for conditions where this would be considered inappropriate. However, a small proportion (32%) of those received antibiotics in the last 7 days before attending the ED. In general, this lack of consistency in antibiotic prescribing highlights the critical need for improved diagnostics and AMS.

Our data suggest that diagnostic uncertainty contributes to inappropriate antibiotic use in viral diseases. While most often the presumed etiology was correct and treated appropriately (Figure 4*A* and 4*B*) when bacterial or viral etiologies were not clearly identified (Figure 4*C*), >50% of cases in the viral group received empiric antibiotics. Since molecular testing often detects both bacterial and viral pathogens in febrile children, it seems difficult for clinicians to withhold antibiotics when a viral cause is identified with the remaining possibility of an additional bacterial infection, while slow diagnostic tools such as cultures are still pending [20]. More than a third of children for whom only viral or noninfectious etiology was recorded as the initial syndrome classification received antibiotics, suggesting that diagnostic uncertainty is not the only driver of inappropriate antibiotic initiation. This effect was particularly seen in the very young: clinicians were more likely to start empiric antibiotics in patients <5 years of age (*P* = .01) (Supplementary Table 9), suggesting that clinicians may be less confident withholding antibiotics in very young febrile children. It was not possible to retrospectively determine whether other factors influenced the decision, such as time of day, social circumstances, parental concerns, or overcrowding.

The Watch antibiotic use for patients within each given final syndrome classification was similar to those with that same initial syndrome classification (Figures 3*A* and 3*C* vs Figure 3*B* and 3*D*), suggesting that in these groups it is not only uncertainty but perhaps other factors such as age and severity of disease that influence clinicians to act cautiously, thus driving excess Watch use. The role of sepsis mandates [21, 22] or fear of missing sepsis and potential litigation may also contribute, at the expense of optimal AMS. The high proportion of Watch antibiotics appears appropriate in some groups, such as central nervous system infections, where third-generation cephalosporins are recommended as first line, or urinary tract infections and intra-abdominal infections caused by gram-negative bacteria with varying resistance profiles.

The most common causative bacteria were *E. coli, S. pyogenes* (group A *Streptococcus*), and *Staphylococcus aureus* and were all associated with considerable empiric Watch antibiotics use. While the resistance pattern of *E. coli* is variable, warranting broader-spectrum antibiotics, this finding is particularly striking for *S. pyogenes,* where often penicillin is a suitable choice [23]. This may reflect the wide variety of syndromes and severity of syndrome associated with this pathogen, ranging from URTIs or soft-tissue infections to severe pneumonia or (toxin-mediated) septic shock.

The most common causative viruses were influenza A/B, rhino/enterovirus, and RSV. More than 60% of patients with RSV and rhino/enterovirus received antibiotics, and overall, 79.7% received Watch antibiotics. Because most of these common viruses can cause sepsislike systemic disease, this may trigger sepsis screening and empiric use of Watch antibiotics [24]. The coronavirus disease 2019 (COVID-19) pandemic has highlighted how sepsislike presentations of viral illness in adult patients can lead to increased use of inappropriate antibiotics [25, 26], showing the pertinence of this phenomenon in the adult setting too.

The strengths of our study are a large prospectively collected multicenter, international cohort over 4 years, stratified by AWaRe classification to characterize antibiotic use. Data from 9 European countries were included, although the largest proportion was recruited from UK centers.

Among the limitations of the study, children recruited in PERFORM are not representative of all febrile children, as only those needing blood tests were recruited; however, diagnostic uncertainty and antibiotic prescribing are likely more relevant in these more severe presentations of illness. In addition, we only used a clearly defined subset of the PERFORM cohort. We did not include patients with a final phenotype of “other infection” ( 27 episodes), “uncertain infection or inflammation” (198 episodes), “inflammatory” (143 episodes) or “trivial” and “other causes of illness” (263 episodes), nor did we include patients categorized as “unknown bacterial or viral” (758 episodes), probable viral (627 episodes), or viral syndrome where there was no viral pathogen identified (193 episodes) [17] (Figure 1), as it would not be possible to consider the appropriateness of antibiotic use in these phenotypes. This skewed our population toward those with a bacterial phenotype, but on the other hand it made the analysis and respective results much clearer.

This data set includes patients with a range of comorbid conditions, some of whom were deemed high risk for infection, and our analysis did not stratify by comorbid condition or by severity of disease. Data on bacterial antibiotic resistance profiles were unavailable, so retrospectively commenting on the appropriateness of using AWaRe antibiotics in view of the actual resistance profile of the detected pathogens was not possible. Data were not available on penicillin allergy status, so antibiotic choices could therefore not be corrected for that.

In conclusion, the differentiation of bacterial or viral etiology of febrile illness on presentation to the ED is challenging. A significant proportion of patients with a final viral phenotype received antibiotics during admission, predominantly classified as Watch. Even when the clinician's judgment suggests a syndrome not requiring antibiotics, clinical uncertainty or concern about a bacterial coinfection or superinfection can result in high Watch antibiotic use until a bacterial cause can be excluded, or a specific pathogen is identified. A recent report from the PERFORM study concluded that it is not always possible to distinguish between bacterial and viral infections, as both pathogens are often jointly detected, leading to broad-spectrum antibiotic use [20]. The tension between AMS and urgent treatment for presumed sepsis is well recognized. However, current guidelines suggest that unless there is septic shock, there is time to wait up to 3 hours for further assessment to decide on the appropriateness of antibiotics [24]. It is here where novel rapid diagnostics could improve AMS, while ensuring that those who need urgent antibiotics receive them.

Future research into improved diagnostic tools is critical for AMS, such as the development of rapid discriminatory point-of-care tests (POCTs). Current POCTs that aid clinicians in differentiating between bacterial and viral infection have limited clinical utility and are not ubiquitously available or favored by clinicians [27]. In some instances, such rapid tools could be useful for improving Access antibiotic use, such as the correct use of rapid antigen testing for *S. pyogenes*, strictly following recommended McIsaac Score assessment [28]. A positive rapid antigen test result may give clinicians confidence to use phenoxymethylpenicillin rather than broader-spectrum alternatives for children presenting with URTIs but would not be as useful for other syndromes caused by this pathogen. Future studies are needed to understand current variability in use and integration of these tests into ED workflow.

Host response–based blood biomarkers can provide reliable prediction of etiology [29]. Clinical trials evaluating the impact of implementing novel host response POCTs on antibiotic prescribing decisions for febrile children in the ED will be crucial. Clinicians worldwide should develop AMS programs that incorporate the AWaRe classification into their strategies, using WHO-defined targets for Access use as a pragmatic framework for monitoring and optimizing antibiotic use. Ultimately, this will enable clinicians worldwide to be more “AWaRe” of the importance of shifting from Watch to Access antibiotic use.

## Supplementary Data

Supplementary materials are available at *Clinical Infectious Diseases* online. Consisting of data provided by the authors to benefit the reader, the posted materials are not copyedited and are the sole responsibility of the authors, so questions or comments should be addressed to the corresponding author.

## Supplementary Material

ciad615_Supplementary_Data

## Notes

***Author Contributions.*** J. H., A. J. C., R. G., V. J. W., M. K., T. K., F. M-T., H. M., M. P., A. J. P., P. K. A. A., L. J. S., M. N. T., S. Y., D. Z., W. Z., M. v. d. F., R. d. G., E. U., F. M., K. F., M. L., E. D. C., M. E., and U. v. B. contributed to the design of the study and funding acquisition. All authors contributed to sample and data collection. T. D. set up, maintained and was primarily responsible for technical aspects of the clinical database, including quality control. P. S. and C. W. were responsible for the database implementation and quality control. L. K. and A. K. were responsible for research-related and clinical quality control of data, performed the statistical analysis and wrote the first draft of the manuscript. L. K., A. K., E. D. C., M. E., and U. v. B. interpreted the data and wrote the final manuscript. All authors have contributed significantly to the drafting and revising of the manuscript and approved the final manuscript. All authors confirm that they had full access to the data and hold responsibility for its content.

***Financial support*.** This project received funding under the European Union’s Horizon 2020 programme (grant agreement 668303) and the Wellcome Trust (grant 203928/Z/16/Z to C. W.).
