# Supplementary material for: Raising AWaRe-ness of Antimicrobial Stewardship Challenges in Pediatric Emergency Care: Results from the PERFORM Study Assessing Consistency and Appropriateness of Antibiotic Prescribing Across Europe
Source: Clin Infect Dis. 2023 Oct 11;78(3):526–34. doi: 10.1093/cid/ciad615 (PMC10954344; doi:10.1093/cid/ciad615)
Supplement: ciad615_Supplementary_Data [file ciad615_supplementary_data.zip › Supplement Perform Consortium List.docx]

**List of PERFORM Consortium Members**

I hereby confirm that the collaborators need to be tagged within the main author list in the online version, and in print they are to appear as a supplementary appendix

**PARTNER: Imperial College, London, UK**

Chief investigator/PERFORM coordinator: Michael Levin^1^

Imperial College: Faculty of Medicine^1^ (in alphabetical order)

Aubrey Cunnington, Tisham De, Jethro A Herberg, Myrsini Kaforou, Victoria J Wright^1^

Imperial College: consortium members^1^ (in alphabetical order)

Lucas Baumard, Evangelos Bellos, Giselle D’Souza, Rachel Galassini, Dominic Habgood-Coote, Shea Hamilton, Clive Hoggart, Sara Hourmat, Heather Jackson, Naomi Lin, Ian Maconochie^4^, Stephanie Menikou, Samuel Nichols, Ruud Nijman, Ivonne Pena Paz, Oliver Powell, Priyen Shah, Ortensia Vito, Clare Wilson

Imperial College: Faculty of Engineering^2^

Molly Stevens (co-investigator), Eunjung Kim, Nayoung Kim

Imperial College Healthcare NHS Trust^3^: clinical recruitment (alphabetical order)

Amina Abdulla, Ladan Ali, Sarah Darnell, Rikke Jorgensen, Sobia Mustafa, Salina Persand

PERFORM UK Clinical Network: Clinical recruitment at Brighton and Sussex University Hospitals^4^

Katy Fidler (principal investigator), Julia Dudley, Vivien Richmond, Emma Tavliavini

Author Affiliations:

1. Section of Paediatric Infectious Disease, Wright-Fleming Institute, Norfolk Place, London W2 1PG, UK
2. Department of Materials, Royal School of Mines, Prince Consort Rd, London SW7 2AZ, UK
3. Children’s Clinical Research Unit, St Mary’s Hospital, Praed Street, London W2 1NY, UK
4. Royal Alexandra Children’s Hospital, Brighton, BN2 5BE, UK.

**PARTNER: University of Liverpool, UK**

Principal Investigator: Enitan D Carrol^1,2,3^

Research Group (in alphabetical order):

Elizabeth Cocklin^1^, Rebecca Jennings^4^, Joanne Johnston^4^, Aakash Khanijau^1^, Simon Leigh^1^, Nadia Lewis-Burke^1^, Karen Newall^4^, Sam Romaine^1^

Author Affiliations:

1. Department of Clinical Infection, Microbiology and Immunology, University of Liverpool Institute of Infection and Global Health, Liverpool, England
2. Alder Hey Children’s Hospital, Department of Infectious Diseases, Eaton Road, Liverpool, L12 2AP
3. Liverpool Health Partners, 1st Floor, Liverpool Science Park, 131 Mount Pleasant, Liverpool, L3 5TF
4. Alder Hey Children’s Hospital, Clinical Research Business Unit, Eaton Road, Liverpool, L12 2AP

**PARTNER: University of Oxford, UK**

Principal Investigators: Andrew J. Pollard^1,2^, Rama Kandasamy^1,2^, Stéphane Paulus^1,2^

Additional Investigators

Michael J. Carter^1,2^, Daniel O'Connor^1,2^, Sagida Bibi^1,2^, Dominic F. Kelly^1,2^, Meeru Gurung^3^, Stephen Thorson^3^, Imran Ansari^3^, David R. Murdoch^4^, Shrijana Shrestha^3^, Zoe Oliver^5^

Author Affiliations:

1. Oxford Vaccine Group, Department of Paediatrics, University of Oxford, Oxford, United Kingdom.
2. NIHR Oxford Biomedical Research Centre, Oxford, United Kingdom.
3. Paediatric Research Unit, Patan Academy of Health Sciences, Kathmandu, Nepal.
4. Department of Pathology, University of Otago, Christchurch, New Zealand.
5. Department of Paediatrics, University of Oxford.

**PARTNER: Newcastle University, Newcastle upon Tyne, UK**

Principal Investigator: Marieke Emonts^1,2,3^

Co-investigators: Emma Lim^2,3,7^, Lucille Valentine^4^

Recruitment team (alphabetical), data-managers, and GNCH Research unit:

Karen Allen^5^, Kathryn Bell^5^, Adora Chan^5^, Stephen Crulley^5^, Kirsty Devine^5^, Daniel Fabian^5^, Sharon King^5^, Paul McAlinden^5^, Sam McDonald^5^, Anne McDonnell^2,5^, Ailsa Pickering^2,5^, Evelyn Thomson^5^, Amanda Wood^5^, Diane Wallia^5^, Phil Woodsford^5^, Frances Baxter^5^, Ashley Bell^5^, Mathew Rhodes^5^

Additional Investigators

Rachel Agbeko^8^, Christine Mackerness^8^, Bryan Baas^2^, Lieke Kloosterhuis^2^, Wilma Oosthoek^2^, Tasnim Arif^6^, Joshua Bennet^2^, Kalvin Collings^2^, Ilona van der Giessen^2^, Alex Martin^2^, Aqeela Rashid^6^, Emily Rowlands^2^, Gabriella de Vries^2^, Fabian van der Velden^2^ , Joshua Soon^2^, Lucille Valentine^4^, Mike Martin^9^, Ravi Mistry^2^, Lucille Valentine^4^

Author Affiliations:

1. Translational and Clinical Research Institute, Newcastle University, Newcastle upon Tyne UK
2. Great North Children’s Hospital, Paediatric Immunology, Infectious Diseases & Allergy, Newcastle upon Tyne Hospitals NHS Foundation Trust, Newcastle upon Tyne, United Kingdom.
3. NIHR Newcastle Biomedical Research Centre based at Newcastle upon Tyne Hospitals NHS Trust and Newcastle University, Westgate Rd, Newcastle upon Tyne NE4 5PL, United Kingdom
4. Newcastle University Business School, Centre for Knowledge, Innovation, Technology and Enterprise (KITE), Newcastle upon Tyne, United Kingdom
5. Great North Children’s Hospital, Research Unit, Newcastle upon Tyne Hospitals NHS Foundation Trust, Newcastle upon Tyne, United Kingdom.
6. Great North Children’s Hospital, Paediatric Oncology, Newcastle upon Tyne Hospitals NHS Foundation Trust, Newcastle upon Tyne, United Kingdom.
7. Population Health Sciences Institute, Newcastle University, Newcastle upon Tyne, UK
8. Great North Children’s Hospital, Paediatric Intensive Care Unit, Newcastle upon Tyne Hospitals NHS Foundation Trust, Newcastle upon Tyne, United Kingdom.
9. Northumbria University, Newcastle upon Tyne, United Kingdom

**PARTNER: London School of Hygiene and Tropical Medicine, UK**

Principal Investigator: Shunmay Yeung^1,2 3^

Research Group

Dr Juan Emmanuel Dewez^1^, Martin Hibberd ^1^, David Bath^2^, Alec Miners^2^, Ruud Nijman^3^, Elizabeth Fitchett

Author Affiliations:

1. Faculty of Infectious and Tropical Disease, London School of Hygiene and Tropical Medicine, London, UK
2. Faculty of Public Health and Policy, London School of Hygiene and Tropical Medicine, London, UK
3. Department of Paediatrics, St. Mary’s Hospital Imperial College Hospital, London, UK
4. Faculty of Epidemiology and Population Health, London School of Hygiene and Tropical Medicine, London, UK

**PARTNER: Micropathology Ltd, Warwick, UK**

Principal Investigator: Colin Fink

Additional investigators

Marie Voice, Leo Calvo-Bado

Author Affiliations:

Micropathology Ltd, The Venture Center, University of Warwick Science Park, Sir William Lyons Road, Coventry, CV4 7EZ

**PARTNER: SERGAS, Santiago de Compostela, Spain**

Principal Investigators: Federico Martinón-Torres^1^, Antonio Salas^1,2^

GENVIP RESEARCH GROUP (in alphabetical order):

Fernando Álvez González^1^, Cristina Balo Farto^1^, Ruth Barral-Arca^1,2^, María Barreiro Castro^1^, Xabier Bello^1,2^, Mirian Ben García^1^, Sandra Carnota^1^, Miriam Cebey-López^1^, María José Curras-Tuala^1,2^, Carlos Durán Suárez^1^, Luisa García Vicente^1^, Alberto Gómez-Carballa^1,2^, Jose Gómez Rial^1^, Pilar Leboráns Iglesias^1^, Federico Martinón-Torres^1^, Nazareth Martinón-Torres^1^, José María Martinón Sánchez^1^, Belén Mosquera Pérez^1^, Jacobo Pardo-Seco^1,2^, Lidia Piñeiro Rodríguez^1^, Sara Pischedda^1,2^, Sara Rey Vázquez^1^, Irene Rivero Calle^1^, Carmen Rodríguez-Tenreiro^1^, Lorenzo Redondo-Collazo^1^, Miguel Sadiki Ora^1^, Antonio Salas^1,2^, Sonia Serén Fernández^1^, Cristina Serén Trasorras^1^, Marisol Vilas Iglesias^1^.

Author Affiliations:

1. Translational Pediatrics and Infectious Diseases, Pediatrics Department, Hospital Clínico Universitario de Santiago, Santiago de Compostela, Spain, and GENVIP Research Group (www.genvip.org), Instituto de Investigación Sanitaria de Santiago, Universidad de Santiago de Compostela, Galicia, Spain.
2. Unidade de Xenética, Departamento de Anatomía Patolóxica e Ciencias Forenses, Instituto de Ciencias Forenses, Facultade de Medicina, Universidade de Santiago de Compostela, and GenPop Research Group, Instituto de Investigaciones Sanitarias (IDIS), Hospital Clínico Universitario de Santiago, Galicia, Spain
3. Fundación Pública Galega de Medicina Xenómica, Servizo Galego de Saúde (SERGAS), Instituto de Investigaciones Sanitarias (IDIS), and Grupo de Medicina Xenómica, Centro de Investigación Biomédica en Red de Enfermedades Raras (CIBERER), Universidade de Santiago de Compostela (USC), Santiago de Compostela, Spain

**PARTNER: Erasmus MC - Sophia Children’s Hospital, Rotterdam, The Netherlands**

Principal Investigators: Henriëtte A. Moll^1^, Clementien L Vermont^2^

Research group

Dorine M. Borensztajn^1^, Nienke N. Hagedoorn^1^, Chantal Tan^1^, Joany Zachariasse^1^, W Dik^3^

Author Affiliations:

1. Erasmus MC-Sophia Children’s Hospital, Department of General Paediatrics, Rotterdam, the Netherlands
2. Erasmus MC-Sophia Children’s Hospital, Department of Paediatric Infectious Diseases & Immunology, Rotterdam, the Netherlands
3. Erasmus MC, Department of immunology, Rotterdam, the Netherlands

**PARTNER: Radboud University Medical Center (RUMC),** **Nijmegen,** **The Netherlands**

Principal Investigators: Ronald de Groot^1^, Michiel van der Flier^1,2,3^, Marien I. de Jonge^1^

Co-investigators Radboud University Medical Center (in alphabetical order):

Koen van Aerde^1,2^, Wynand Alkema^1^, Bryan van den Broek^1^, Jolein Gloerich^1^, Alain J. van Gool^1^, Stefanie Henriet^1,2^, Martijn Huijnen^1^, Ria Philipsen^1^, Esther Willems^1^

Investigators PeDBIG PERFORM DUTCH CLINICAL NETWORK (in alphabetical order):

G.P.J.M. Gerrits^8^, M. van Leur^8^, J. Heidema ^4^, L. de Haan^1,2^, C.J. Miedema ^5^, C. Neeleman^1^, C.C. Obihara^6^, G.A. Tramper-Stranders^6,7^

Author Affiliations:

1. Radboud University Medical Center, Nijmegen, The Netherlands
2. Amalia Children’s Hospital, Nijmegen, The Netherlands
3. Wilhelmina Children’s Hospital, University Medical Center Utrecht, Utrecht, The Netherlands
4. St. Antonius Hospital, Nieuwegein, The Netherlands
5. Catharina Hospital, Eindhoven, The Netherlands
6. ETZ Elisabeth, Tilburg, The Netherlands
7. Franciscus Gasthuis, Rotterdam, The Netherlands
8. Canisius Wilhelmina Hospital, Nijmegen, The Netherlands

**PARTNER: Academic Medical Hospital & Sanquin Research Institute, Amsterdam, The Netherlands**

Principal Investigator: Taco Kuijpers^1,2^

Co-investigators: Ilse Jongerius^2^

Recruitment team:

J.M. van den Berg^1^, D. Schonenberg^1^, A.M. Barendregt^1^, D. Pajkrt^1^, M. van der Kuip^1,3^, A.M. van Furth^1,3^

Students and technical support:

Evelien Sprenkeler^2^, Judith Zandstra^2^, G. van Mierlo^2^, J. Geissler^2^

Author Affiliations:

1. Amsterdam University Medical Center (Amsterdam UMC), location Academic Medical Center (AMC), Dept of Pediatric Immunology, Rheumatology and Infectious Diseases, University of Amsterdam, Amsterdam, the Netherlands
2. Sanquin Research Institute, & Landsteiner Laboratory at the AMC, University of Amsterdam, Amsterdam, the Netherlands.
3. Amsterdam University Medical Center (Amsterdam UMC), location Vrije Universiteit Medical Center (VUMC), Dept of Pediatric Infectious Diseases and Immunology, Free University (VU), Amsterdam, the Netherlands (former affiliation)

**PARTNER: Riga Stradins University, Latvia**

Principal Investigator: Dace Zavadska^1,2^

Other RSU group authors (in alphabetical order):

Anda Balode^1,2^, Arta Bārzdiņa^1,2^, Dārta Deksne^1,2^, Dace Gardovska^1,2^, Dagne Grāvele^2^, Ilze Grope^1,2^, Anija Meiere^1,2^, Ieva Nokalna^1,2^, Jana Pavāre^1,2^, Zanda Pučuka^1,2^, Katrīna Selecka^1,2^, Aleksandra Rudzāte^1,2^, Dace Svile^2^, Urzula Nora Urbāne^1,2^

Author Affiliations:

1. Riga Stradins university, Riga, Latvia.
2. Children clinical university hospital, Riga, Latvia.

**PARTNER: Medical University of Graz (MUG), Austria**

Principal Investigator: Werner Zenz^1^

Co-investigators (in alphabetical order)

Benno Kohlmaier^1^, Nina A. Schweintzger^1^, Manfred G. Sagmeister^1^

Research team

Daniela S. Kohlfürst^1^, Christoph Zurl^1^, Alexander Binder^1^

Recruitment team, data managers, (in alphabetical order):

Susanne Hösele^1^, Manuel Leitner^1^, Lena Pölz^1^, Glorija Rajic^1^,

Clinical recruitment partners (in alphabetical order):

Sebastian Bauchinger^1^, Hinrich Baumgart^4^, Martin Benesch^3^, Astrid Ceolotto^1^, Ernst Eber^2^, Siegfried Gallistl^1^, Gunther Gores^5^, Harald Haidl^1^, Almuthe Hauer^1^, Christa Hude^1^, Markus Keldorfer^5^, Larissa Krenn^4^, Heidemarie Pilch^5^, Andreas Pfleger^2^, Klaus Pfurtscheller^4^, Gudrun Nordberg^5^, Tobias Niedrist^8^, Siegfried Rödl^4^, Andrea Skrabl-Baumgartner^1^, Matthias Sperl^7^, Laura Stampfer^5^, Volker Strenger^3^, Holger Till^6^, Andreas Trobisch^5^, Sabine Löffler^5^

Author Affiliations:

1. Department of Pediatrics and Adolescent Medicine, Division of General Pediatrics, Medical University of Graz, Graz, Austria
2. Department of Pediatric Pulmonology, Medical University of Graz, Graz, Austria
3. Department of Pediatric Hematooncoloy, Medical University of Graz, Graz, Austria
4. Paediatric Intensive Care Unit, Medical University of Graz, Graz, Austria
5. University Clinic of Paediatrics and Adolescent Medicine Graz, Medical University Graz, Graz,Austria
6. Department of Paediatric and Adolescence Surgery, Medical University Graz, Graz, Austria
7. Department of Pediatric Orthopedics, Medical University Graz, Graz, Austria
8. Clinical Institute of Medical and Chemical Laboratory Diagnostics, Medical University Graz, Graz, Austria

**PARTNER: Hauner Children’s Hospital, LMU Munich, Germany**

Principal Investigator: Ulrich von Both^1,2^

Research group:

Laura Kolberg¹, Manuela Zwerenz¹, Judith Buschbeck¹

Clinical recruitment partners (in alphabetical order):

Christoph Bidlingmaier^3^, Vera Binder^4^, Katharina Danhauser^5^, Nikolaus Haas^10^, Matthias Griese^6^, Tobias Feuchtinger^4^, Julia Keil^9^, Matthias Kappler^6^, Eberhard Lurz^7^, Georg Muench^8^, Karl Reiter^9^, Carola Schoen^9^

Author Affiliations:

1. Div. Paediatric Infectious Diseases, Hauner Children’s Hospital, University Hospital, Ludwig Maximilians University (LMU), Munich, Germany
2. German Center for Infection Research (DZIF), Partner Site Munich, Munich, Germany
3. Div. of General Paediatrics,
4. Div. Paediatric Haematology & Oncology,
5. Div. of Paediatric Rheumatology,
6. Div. of Paediatric Pulmonology,
7. Div. of Paediatric Gastroenterology,
8. Neonatal Intensive Care Unit,
9. Paediatric Intensive Care Unit Hauner Children’s Hospital,
10. Department Pediatric Cardiology and Pediatric Intensive Care, University Hospital, LMU, Munich, Germany

**PARTNER: National and Kapodistrian University of Athens (NKUA), Greece**

Principal investigator: Maria Tsolia

Investigator/Research fellow: Irini Eleftheriou

Additional investigators:

Maria Tambouratzi, Antonis Marmarinos, Marietta Xagorari, Kelly Syggelou

Author Affiliations:

2nd Department of Pediatrics, National and Kapodistrian University of Athens, “P. and A. Kyriakou” Children’s Hospital, Thivon and Levadias, Goudi, Athens

**PARTNER: Swiss Pediatric Sepsis Study, Switzerland**

Principal Investigators: Philipp Agyeman^1^, Luregn J Schlapbach^2,3^

Clinical recruitment at University Children’s Hospital Bern for PERFORM:

Christoph Aebi^1^, Verena Wyss^1^, Mariama Usman^1^

Principal and co-investigators for the Swiss Pediatric Sepsis Study:

Eric Giannoni^4,5^, Martin Stocker^6^, Klara M Posfay-Barbe^7^, Ulrich Heininger^8^, Sara Bernhard-Stirnemann^9^, Anita Niederer-Loher^10^, Christian Kahlert^10^, Giancarlo Natalucci^11^, Christa Relly^12^, Thomas Riedel^13^, Christoph Aebi^1^, Christoph Berger^12^

Author Affiliations:

1. Department of Pediatrics, Inselspital, Bern University Hospital, University of Bern, Switzerland
2. Neonatal and Pediatric Intensive Care Unit, Children’s Research Center, University Children’s Hospital Zurich, University of Zurich, Zurich, Switzerland
3. Child Health Research Centre, University of Queensland, and Queensland Children`s Hospital, Brisbane, Australia
4. Clinic of Neonatology, Department Mother-Woman-Child, Lausanne University Hospital and University of Lausanne, Switzerland
5. Infectious Diseases Service, Department of Medicine, Lausanne University Hospital and University of Lausanne, Switzerland
6. Department of Pediatrics, Children’s Hospital Lucerne, Lucerne, Switzerland
7. Pediatric Infectious Diseases Unit, Children’s Hospital of Geneva, University Hospitals of Geneva, Geneva, Switzerland
8. Infectious Diseases and Vaccinology, University of Basel Children’s Hospital, Basel, Switzerland
9. Children’s Hospital Aarau, Aarau, Switzerland
10. Division of Infectious Diseases and Hospital Epidemiology, Children’s Hospital of Eastern Switzerland St. Gallen, St. Gallen, Switzerland
11. Department of Neonatology, University Hospital Zurich, Zurich, Switzerland
12. Division of Infectious Diseases and Hospital Epidemiology, and Children’s Research Center, University Children’s Hospital Zurich, Switzerland
13. Children’s Hospital Chur, Chur, Switzerland

**PARTNER: Department of Infectious Diseases, University Medical Centre Ljubljana, Slovenia**

Principal Investigator: Marko Pokorn^1,2,3^

Research Group:

Mojca Kolnik^1^, Katarina Vincek^1^, Tina Plankar Srovin^1^, Natalija Bahovec^1^, Petra Prunk^1^, Veronika Osterman^1^, Tanja Avramoska^1^

Author Affiliations:

1. Department of Infectious Diseases, University Medical Centre Ljubljana, Japljeva 2, SI-1525 Ljubljana, Slovenia
2. University Childrens' Hospital, University Medical Centre Ljubljana, Ljubljana, Slovenia
3. Department of Infectious Diseases and Epidemiology, Faculty of Medicine, University of Ljubljana, Slovenia

**PARTNER: bioMérieux, France**

Principal Investigator: François Mallet^1,2, 3^

Research Group:

Karen Brengel-Pesce^1,2, 3^, Alexandre Pachot^1^, Marine Mommert^1,2^

Author Affiliations:

1. Open Innovation & Partnerships (OIP), bioMérieux S.A., Marcy l'Etoile, France
2. Joint research unit Hospice Civils de Lyon - bioMérieux, Centre Hospitalier Lyon Sud, 165 Chemin du Grand Revoyet, 69310 Pierre-Bénite, France
3. EA 7426 Pathophysiology of Injury-induced Immunosuppression, University of Lyon1-Hospices Civils de Lyon-bioMérieux, Hôpital Edouard Herriot, 5 Place d’Arsonval, 69437 Lyon Cedex 3, France

**PARTNER: Medical Research Council Unit The Gambia (MRCG) at LSHTM**

Principal Investigator: Effua Usuf

Additional Investigators

Kalifa Bojang, Syed M. A. Zaman, Fatou Secka, Suzanne Anderson, Anna RocaIsatou Sarr, Momodou Saidykhan, Saffiatou Darboe, Samba Ceesay, Umberto D’alessandro

Author Affiliations:

Medical Research Council Unit The Gambia at LSHTM, P O Box 273, Fajara, The Gambia

**PARTNER: National Cheng Kung University Hospital, Taiwan**

Principal Investigator: Ching-Fen Shen

Co-investigator: Ching-Chuan Liu, Shih-Min Wang

funded by the Center of Clinical Medicine Research, National Cheng Kung University
