# Supplementary material for: Raising AWaRe-ness of Antimicrobial Stewardship Challenges in Pediatric Emergency Care: Results from the PERFORM Study Assessing Consistency and Appropriateness of Antibiotic Prescribing Across Europe
Source: Clin Infect Dis. 2023 Oct 11;78(3):526–34. doi: 10.1093/cid/ciad615 (PMC10954344; doi:10.1093/cid/ciad615)
Supplement: ciad615_Supplementary_Data [file ciad615_supplementary_data.zip › SUPPLEMENT_revised_CID_2023_09_01_final.docx]

**Raising AWaRe-ness of antimicrobial stewardship challenges in paediatric emergency care: results from the PERFORM study assessing consistency and appropriateness of antibiotic prescribing across Europe**

**Supplementary material**

**Table of contents**

[Supplementary Table 1 2](#_Toc144469715)

[Supplementary Table 2 3](#_Toc144469716)

[Supplementary Table 3 3](#_Toc144469717)

[Supplementary Table 4 3](#_Toc144469718)

[Supplementary Table 5 4](#_Toc144469719)

[Supplementary Table 6 5](#_Toc144469720)

[Supplementary Table 7 6](#_Toc144469721)

[Supplementary Table 8 7](#_Toc144469722)

[Supplementary Table 9 8](#_Toc144469723)

[Supplementary Table 10 8](#_Toc144469724)

[Supplementary Table 11 9](#_Toc144469725)

[Supplementary Table 12 10](#_Toc144469726)

[Supplementary Figure 1 11](#_Toc144469727)

[Supplementary Figure 2 12](#_Toc144469728)

| **Supplementary Table 1:** Ethic committees of the participating sites | | |
| --- | --- | --- |
| **Country** | **Partner** | **Ethics** |
| United Kingdom | Imperial College of Science, Technology and Medicine, Section of Paediatrics, Wright-Fleming Institute  Chief investigator/PERFORM coordinator: Michael Levin  Principal Investigators: Jethro Herberg  Clinical recruitment at Brighton and Sussex University Hospitals  Principal Investigator: Katy Fidler  The University of Liverpool Institute of Infection and Global Health, Department of Clinical Infection, Microbiology and Immunology  Principal Investigator: Enitan D Carrol  London School of Hygiene and Tropical Medicine, Department of Clinical Research  Faculty of Tropical and Infectious Disease  Principal Investigator: Shunmay Yeung  John Radcliffe Hospital Oxford  Principal Investigators: Andrew J. Pollard, Rama Kandasamy, Stéphane Paulus  University of Newcastle Upon Tyne: Newcastle upon Tyne Hospitals NHS Foundation Trust, Great North Children’s Hospital  Paediatric Immunology, Infectious Diseases & Allergy  Principal Investigator: Marieke Emonts | United Kingdom (Ethics Committee, ID: 16/LO/1684, IRAS application no. 209035, Confidentiality advisory group reference: 16/CAG/0136). |
| Spain | Servizo Galego de Saude SERGAS  Hospital Clínico Universitario de Santiago de Compostela (CHUS)- Spain  Genetics, Vaccines, Infections and Pediatrics Research group (GENVIP)  Principal Investigator: Federico Martinón-Torres | Spain (Comité Autonómico de Ética de la Investigación de Galicia, ID: 2016/331) |
| Latvia | Rīgas Stradiņa universitāte (RSU), Department of Pediatrics  Children clinical university hospital  Principal Investigator: Dace Zavadska | Latvia (Centrala medicinas etikas komiteja, ID: 14.07.201 6. No. Il 16-07 -14) |
| The Netherlands | ERASMUS universitair medisch centrum Rotterdam, Sophia’s Childrens Hospital  Principal Investigators: Henriëtte A. Moll, Clementien L Vermont  Academic Medical Hospital & Sanquin Research Institute, Amsterdam  Principal Investigator: Taco Kuijpers  Radboud University Medical Center (RUMC) Stichting Katholieke Universiteit  Principal Investigators: Ronald de Groot, Michiel van der Flier, Marien I. de Jonge | The Netherlands (Commissie Mensgebonden onderzoek, ID: NL58103.091.16) |
| Switzerland | University Bern, Children Hospital Department of Pediatrics  Principal Investigators: Philipp Agyeman, Luregn J Schlapbach | Kantonale Ethikkommission Bern, KEK-Gesuchs-Nr.: 029/11 |
| Greece | National and Kapodistrian University of Athens (NKUA), Second Department of Paediatrics,  Principal investigator: Professor Maria Tsolia | Greece (Ethics committee, ID: 9683/18.07.2016) |
| Austria | Medical University of Graz, (MUG), Department of General Paediatrics  Principal Investigator: Werner Zenz | Austria (Ethikkommission Medizinische Universitat Graz, ID: 28-518 ex 15/16) |
| Germany | Ludwig-Maximilian-University Munich (LMU), Division of Paediatric Infectious Diseases  Principal Investigator: Ulrich von Both | Germany (Ethikkommission der LMU München, ID: 699-16) |
| Slovenia | University Medical Centre Ljubljana, Department of Infectious Diseases  Principal Investigator: Marko Pokorn | Slovenia (Republic of Slovenia National Medical Ethics Committee, ID: 0120-483/2016- 3) |

| **Supplementary Table 2: List of the syndrome classification on the PERFORM CRF** |  |
| --- | --- |
| **Syndrome classification** |  |
| LOWER RESPIRATORY TRACT INFECTION (LRTI) | Main syndromes |
| UPPER RESPIRATORY TRACT INFECTION / EAR, NOSE, THROAT (URTI/ENT) |  |
| MUSKULOSKELETAL |  |
| CENTRAL NERVOUS SYSTEM INFECTION |  |
| GASTROINTESTINAL INFECTION / SURGICAL OR INTRA-ABDOMINAL INFECTION |  |
| SOFT TISSUE INFECTION |  |
| URINARY TRACT INFECTION |  |
| PATHOGEN SYNDROME |  |
| SEPSIS SYNDROME |  |
| UNDIFFERENTIATED FEVER |  |
| FEBRILE NEUTROPENIA |  |
| OTHER INFECTIONS |  |
| For the purposes of analysis a number of changes were made:  - GASTROINTESTINAL INFECTION / SURGICAL OR INTRA-ABDOMINAL INFECTION was split into GastrointestinaI (GI) and surgical/intra-abdominal infection (SURG/INTRA-ABDO)  - PATHOGEN SYNDROME was split into (Bacterial pathogen syndrome, Viral pathogen syndrome and Other pathogen syndrome)  - SEPSIS SYNDROME was re-named Sepsis syndrome/Endovascular infection (SEPSIS/ENDO) to avoid confusion with separate analysis of patients meeting ‘Goldstein criteria’  - Febrile neutropenia and Neutropenic sepsis were separated into their own category: neutropenia  **-** Inflammatory syndrome was created | |

| **Supplementary Table 3: Empiric antibiotic classes and AWaRe classes prescribed in our dataset** | |
| --- | --- |
| Access | Cefadroxil, Cefalexin, Cefazolin, Amikacin, Gentamicin, Chloramphenicol, Trimethoprim, Trimethoprim Sulfamethoxazole (co-trimoxazole), Metronidazole, Clindamycin, Nitrofurantoin, Ampicillin/sulbactam, Co-amoxiclav (Augmentin, amoxicillin-clavulanate), Amoxicillin, Ampicillin, Benzlypenicillin (Pencillin G), Cloxacillin, Flucloxacillin, Phenoxymethylpenicillin (Penicillin V), Doxycycline |
| Watch | Cefaclor, Cefuroxime, Cefixime, Cefotaxime, Ceftazidime, Ceftriaxone, Cefepime, Meropenem, Ciprofloxacin, Teicoplanin, Vancomycin, Azithromycin, Clarithromycin, Erythromycin, Piperacillin/tazobactam, Rifampicin, Tobramycin, Ofloxacin |
| Reserve | Linezolid |
| 3 antibiotics were unclassified (Ethambutol, Isoniazid and Pyrazinamide) | |

| **Supplementary Table 4 Definition of antibiotic use** | |
| --- | --- |
| CONSISTENT antibiotic use | presumed viral/ non-infectious etiology + NO antibiotics presumed bacterial etiology + antibiotics |
| INCONSISTENT antibiotic use | presumed viral/ non-infectious + antibiotics presumed bacterial etiology + NO antibiotics |
| APPROPRIATE antibiotic use | final viral phenotype + NO antibiotics  final bacterial phenotype + antibiotics * |
| INAPPROPRIATE antibiotic use | final viral phenotype + antibiotics final bacterial phenotype + NO antibiotics * |
| *unless certain diagnoses, defined in Supplementary Table 5 | |

| **Supplementary Table 5: 231 patients with final bacterial phenotype in whom no empiric antibiotics are initiated in the first 48 hours (n)** | | | | | | |
| --- | --- | --- | --- | --- | --- | --- |
| **Final syndrome classification** | **Diagnoses within syndrome classification that were considered appropriate to withhold antibiotics for:** | **Total number**  **(231)** | **Appropriate**  **(81)** | **Inappropriate**  **(120)** | **Unable to judge**  **(29)** | **Condition warranting antibiotics, that had antibiotics in last 7 days (39)** |
| LRTI | Undefined LRTI, Bronchitis, Bronchiolitis, Pulmonary TB | 37 | 3 | 34 | 0 | 6 |
| URTI | Otitis Media, Tonsillitis/Pharyngitis, URTI non specific, Stomatitis | 31 | 25 | 0 | 6* |  |
| MSK | - | 4 | 0 | 4 | 0 | 2 |
| CNS | - | 3 | 0 | 3 | 0 | 0 |
| GI | Gastroenteritis with any bacterial pathogen other than *C·difficile* | 46 | 46 | 0 | 0 |  |
| SURG/INTRA-ABDO | Mesenteric adenitis | 19 | 0 | 1 | 19** | 0 |
| SOFT TISSUE | Soft tissue abscess | 18 | 2 | 15 | 1*** | 12 |
| UTI | - | 31 | 0 | 31 | 0 | 13 |
| VPS | Any | 0 |  |  |  |  |
| BPS | - | 1 | 0 | 1 | 0 | 0 |
| SEPSIS/ENDO | - | 6 | 0 | 6 | 0 | 0 |
| UNDIFF FEVER | Febrile convulsion, Fever without source | 0 |  |  |  |  |
| OTHER | Conjunctivitis | 0 | 0 | 0 | 0 |  |
| LRTI + URTI | Included the above diagnoses for individual syndrome classifications | 2 | 1 | 1 | 0 | 0 |
| LRTI + SEPSIS/ENDO |  | 2 | 0 | 2 | 0 | 0 |
| LRTI + OTHER |  | 2 | 1 | 1 | 0 | 0 |
| LRTI + VPS |  | 1 | 0 | 1 | 0 | 0 |
| URTI + GI |  | 4 | 4 | 0 | 0 | 0 |
| URTI + SURG/INTRA-ABDO |  | 1 | 0 | 0 | 1** |  |
| URTI + CNS + OTHER |  | 1 | 0 | 1 | 0 | 0 |
| URTI+SOFT TISSUE |  | 1 | 0 | 1 | 0 | 1 |
| URTI+VPS |  | 1 | 1 | 0 | 0 |  |
| URTI+BPS |  | 1 | 0 | 1 | 0 | 0 |
| URTI+SOFT TISSUE + UNDIFF FEVER |  | 1 | 0 | 1 | 0 | 0 |
| MSK+GI |  | 1 | 0 | 1 | 0 | 0 |
| MSK+SEPSIS/ENDO |  | 4 | 0 | 4 | 0 | 1 |
| CNS+BPS |  | 1 | 0 | 1 | 0 | 0 |
| CNS+SEPSIS/ENDO |  | 3 | 0 | 3 | 0 | 0 |
| GI + SEPSIS/ENDO |  | 1 | 0 | 1 | 0 | 0 |
| SURG/INTRA-ABDO+VPS |  | 1 | 0 | 0 | 1** |  |
| SOFT TISSUE+SEPSIS/ENDO |  | 1 | 0 | 1 | 0 | 1 |
| SOFT TISSUE+VPS |  | 2 | 0 | 1 | 1*** | 1 |
| UTI+SEPSIS/ENDO |  | 2 | 0 | 2 | 0 | 0 |
| SEPSIS/ENDO+UNDIFF FEVER+OTHER |  | 1 | 0 | 1 | 0 | 1 |
| LRTI+URTI+SEPSIS/ENDO |  | 1 | 0 | 1 | 0 | 1 |
| *Timing of onset of bacterial infection unclear in relation to presentation to hospital  **Varying policy by country re: antibiotic use in children with appendicitis  ***Received topical antibiotics  LRTI: lower respiratory tract infection  URTI: upper respiratory tract infection, ear nose throat  CNS: central nervous system infection  MSK: musculoskeletal infection  GI: gastrointestinal infection  BPS: bacterial pathogen syndrome  VPS: viral pathogen syndrome  SEPSIS/ENDO: sepsis syndrome/endovascular infection  SURG/INTRA-ABDO: surgical/intra-abdominal infection  UTI: urinary tract infection  SOFT TISSUE: soft tissue infection  UNDIFF FEVER: undifferentiated Fever | | | | | | |

| **Supplementary Table 6**: **Spectrum of initial and final diagnoses in ‘bacterial’ and ‘viral’ group (n; %)** | | | | | | | | | | | | |
| --- | --- | --- | --- | --- | --- | --- | --- | --- | --- | --- | --- | --- |
|  | initial diagnosis | | | | | | final diagnosis | | | | | |
|  | ‘bacterial’ group | | ‘viral’ group | | total | | ‘bacterial’ group | | ‘viral’ group | | total | |
| LRTI | 289 | 18·7 | 132 | 22·7 | 421 | 19·8 | 357 | 23·0 | 144 | 24·8 | 501 | 23·5 |
| URTI/EAR NOSE THROAT | 283 | 18·3 | 116 | 20·0 | 399 | 18·7 | 292 | 18·9 | 130 | 22·4 | 425 | 20·0 |
| SOFT TISSUE INFECTION | 298 | 19·2 | 16 | 2·8 | 314 | 14·7 | 321 | 20·7 | 9 | 1·5 | 330 | 15·5 |
| UNDIFFERENTIATED FEVER | 180 | 11·6 | 72 | 12·4 | 252 | 11·8 | 25 | 1·6 | 28 | 4·8 | 53 | 2·5 |
| URINARY TRACT INFECTION | 202 | 13·0 | 4 | 0·7 | 206 | 9·7 | 272 | 17·6 | 1 | 0·2 | 274 | 12·9 |
| VIRAL PATHOGEN SYNDROME | 59 | 3·8 | 146 | 25·1 | 205 | 9·6 | 44 | 2·8 | 224 | 38·6 | 268 | 12·6 |
| GASTROINTESTINAL INFECTION | 121 | 7·8 | 57 | 9·8 | 178 | 8·4 | 95 | 6·1 | 58 | 10·0 | 153 | 7·2 |
| SEPSIS/ENDO | 116 | 7·5 | 42 | 7·2 | 158 | 7·4 | 189 | 12·2 | 2 | 0·3 | 191 | 9·0 |
| SURG/INTRA-ABDO | 119 | 7·7 | 6 | 1·0 | 125 | 5·9 | 116 | 7·5 | 1 | 0·2 | 117 | 5·5 |
| CNS INFECTION | 38 | 2·5 | 44 | 7·6 | 82 | 3·8 | 44 | 2·8 | 57 | 9·8 | 101 | 4·7 |
| MUSCULOSKELETAL INFECTION | 60 | 3·9 | 13 | 2·2 | 73 | 3·4 | 53 | 3·4 | 15 | 2·6 | 68 | 3·2 |
| OTHER | 37 | 2·4 | 18 | 3·1 | 55 | 2·6 | 25 | 1·6 | 14 | 2·4 | 39 | 1·8 |
| BACTERIAL PATHOGEN SYNDROME | 35 | 2·3 | 2 | 0·3 | 37 | 1·7 | 47 | 3·0 | 0 | 0 | 47 | 2·2 |
| FEBRILE NEUTROPENIA | 14 | 0·9 | 10 | 1·7 | 24 | 1·1 | 6 | 0·4 | 2 | 0·3 | 8 | 0·4 |
| INFLAMMATORY SYNDROME | 9 | 0·6 | 7 | 1·2 | 16 | 0·8 | 0 | 0 | 0 | 0 | 0 | 0 |
| OTHER PATHOGEN SYNDROME | 4 | 0·3 | 4 | 0·7 | 8 | 0·4 | 3 | 0·2 | 0 | 0 | 3 | 0·1 |
| UNKNOWN | 5 | 0·3 | 1 | 0·2 | 6 | 0·3 | 0 | 0 | 0 | 0 | 0 | 0 |
| LRTI: lower respiratory tract infection | | | | | | | | | | | | |
| URTI: upper respiratory tract infection | | | | | | | | | | | | |
| CNS: central nervous system | | | | | | | | | | | | |
| SURG/INTRA-ABDO: surgical/intra-abdominal infection | | | | | | | | | | | | |
| SEPSIS/ENDO: sepsis syndrome/endovascular infection | | | | | | | | | | | | |

| **Supplementary Table 7: Total antibiotic use by country in ‘bacterial’ group (n; %)** | | | | | | | | | | | | | | | | | | | | |
| --- | --- | --- | --- | --- | --- | --- | --- | --- | --- | --- | --- | --- | --- | --- | --- | --- | --- | --- | --- | --- |
| Country (n) | Austria | | Germany | | Greece | | Latvia | | Slovenia | | Spain | | Switzerland | | Netherlands | | United Kingdom | | Total | |
|  | 148 | | 21 | | 149 | | 194 | | 127 | | 152 | | 79 | | 186 | | 493 | | 1549 | |
| Received systemic antibiotics | 121 | 81·8 | 15 | 71·4 | 140 | 94·0 | 178 | 91·8 | 112 | 88·2 | 54 | 35·5 | 67 | 84·8 | 169 | 90·9 | 462 | 93.7 | 1318 | 85.1 |
| **ALL % BELOW IS OUT OF THOSE WHO RECEIVED SYSTEMIC ANTIBIOTICS** | | | | | | | | | | | | | | | | | | | | |
| IV/IM* antibiotics | 103 | 85.1 | 10 | 66.7 | 127 | 90.7 | 153 | 86.0 | 104 | 92.9 | 39 | 72.2 | 56 | 83.6 | 137 | 81.1 | 428 | 92.6 | 1157 | 87.8 |
| First Generation Cephalosporins | 4 | 3.3 | 0 | 0.0 | 0 | 0.0 | 3 | 1.7 | 3 | 2.7 | 0 | 0.0 | 0 | 0.0 | 2 | 1.2 | 40 | 8.7 | 52 | 3.9 |
| Second Generation Cephalosporins | 32 | 26.4 | 6 | 40.0 | 23 | 16.4 | 67 | 37.6 | 4 | 3.6 | 4 | 7.4 | 26 | 38.8 | 22 | 13.0 | 24 | 5.2 | 208 | 15.8 |
| Third Generation Cephalosporins | 11 | 9.1 | 3 | 20.0 | 53 | 37.9 | 39 | 21.9 | 8 | 7.1 | 25 | 46.3 | 8 | 11.9 | 58 | 34.3 | 251 | 54.3 | 456 | 34.6 |
| Fourth Generation Cephalosporins | 3 | 2.5 | 0 | 0.0 | 5 | 3.6 | 0 | 0.0 | 0 | 0.0 | 0 | 0.0 | 3 | 4.5 | 0 | 0.0 | 0 | 0.0 | 11 | 0.8 |
| Aminoglycoside | 0 | 0.0 | 0 | 0.0 | 22 | 15.7 | 8 | 4.5 | 16 | 14.3 | 2 | 3.7 | 3 | 4.5 | 25 | 14.8 | 57 | 12.3 | 133 | 10.1 |
| Carbapenems | 4 | 3.3 | 3 | 20.0 | 4 | 2.9 | 0 | 0.0 | 1 | 0.9 | 4 | 7.4 | 0 | 0.0 | 2 | 1.2 | 16 | 3.5 | 34 | 2.6 |
| DHFR inhibitors | 2 | 1.7 | 0 | 0.0 | 0 | 0.0 | 2 | 1.1 | 9 | 8.0 | 0 | 0.0 | 1 | 1.5 | 6 | 3.6 | 3 | 0.6 | 23 | 1.7 |
| Fluoroquinolones | 1 | 0.8 | 0 | 0.0 | 1 | 0.7 | 3 | 1.7 | 0 | 0.0 | 0 | 0.0 | 2 | 3.0 | 8 | 4.7 | 18 | 3.9 | 33 | 2.5 |
| Glycopeptides | 5 | 4.1 | 0 | 0.0 | 9 | 6.4 | 5 | 2.8 | 1 | 0.9 | 3 | 5.6 | 1 | 1.5 | 13 | 7.7 | 37 | 8.0 | 74 | 5.6 |
| Imidazoles | 1 | 0.8 | 1 | 6.7 | 2 | 1.4 | 20 | 11.2 | 0 | 0.0 | 0 | 0.0 | 14 | 20.9 | 12 | 7.1 | 57 | 12.3 | 107 | 8.1 |
| Lincosamides | 10 | 8.3 | 0 | 0.0 | 23 | 16.4 | 17 | 9.6 | 3 | 2.7 | 4 | 7.4 | 6 | 9.0 | 10 | 5.9 | 46 | 10.0 | 119 | 9.0 |
| Macrolides | 7 | 5.8 | 0 | 0.0 | 6 | 4.3 | 26 | 14.6 | 2 | 1.8 | 5 | 9.3 | 2 | 3.0 | 6 | 3.6 | 52 | 11.3 | 106 | 8.0 |
| Nitrofurantoin | 0 | 0.0 | 1 | 6.7 | 0 | 0.0 | 0 | 0.0 | 0 | 0.0 | 0 | 0.0 | 0 | 0.0 | 1 | 0.6 | 1 | 0.2 | 3 | 0.2 |
| Oxazolidinones | 1 | 0.8 | 0 | 0.0 | 1 | 0.7 | 0 | 0.0 | 0 | 0.0 | 0 | 0.0 | 1 | 1.5 | 0 | 0.0 | 2 | 0.4 | 5 | 0.4 |
| Penicillin/Beta-lactamase Inhibitor Combinations | 67 | 55.4 | 5 | 33.3 | 9 | 6.4 | 5 | 2.8 | 38 | 33.9 | 10 | 18.5 | 19 | 28.4 | 63 | 37.3 | 194 | 42.0 | 410 | 31.1 |
| Penicillins | 12 | 9.9 | 2 | 13.3 | 46 | 32.9 | 51 | 28.7 | 52 | 46.4 | 16 | 29.6 | 7 | 10.4 | 47 | 27.8 | 121 | 26.2 | 354 | 26.9 |
| Rifamycins | 1 | 0.8 | 0 | 0.0 | 1 | 0.7 | 0 | 0.0 | 0 | 0.0 | 0 | 0.0 | 0 | 0.0 | 0 | 0.0 | 1 | 0.2 | 3 | 0.2 |
| Tetracyclines | 0 | 0.0 | 0 | 0.0 | 0 | 0.0 | 1 | 0.6 | 0 | 0.0 | 0 | 0.0 | 1 | 1.5 | 0 | 0.0 | 0 | 0.0 | 2 | 0.2 |
| Other** | 1 | 0.8 | 0 | 0.0 | 0 | 0.0 | 10 | 5.6 | 1 | 0.9 | 0 | 0.0 | 1 | 1.5 | 2 | 1.2 | 2 | 0.4 | 17 | 1.3 |
| Unknown | 0 | 0.0 | 0 | 0.0 | 0 | 0.0 | 0 | 0.0 | 1 | 0.9 | 0 | 0.0 | 0 | 0.0 | 0 | 0.0 | 0 | 0.0 | 1 | 0.1 |
| 1 Access | 77 | 63.6 | 7 | 46.7 | 70 | 50.0 | 79 | 44.4 | 64 | 57.1 | 30 | 55.6 | 42 | 62.7 | 78 | 46.2 | 255 | 55.2 | 702 | 53.3 |
| 2 Access | 9 | 7.4 | 0 | 0.0 | 14 | 10.0 | 14 | 7.9 | 38 | 33.9 | 1 | 1.9 | 4 | 6.0 | 30 | 17.8 | 85 | 18.4 | 195 | 14.8 |
| 3 Access | 0 | 0.0 | 0 | 0.0 | 1 | 0.7 | 0 | 0.0 | 0 | 0.0 | 0 | 0.0 | 0 | 0.0 | 8 | 4.7 | 13 | 2.8 | 22 | 1.7 |
| 4 Access | 0 | 0.0 | 0 | 0.0 | 0 | 0.0 | 0 | 0.0 | 0 | 0.0 | 0 | 0.0 | 0 | 0.0 | 0 | 0.0 | 3 | 0.6 | 3 | 0.2 |
| At least one Access | 86 | 71.1 | 7 | 46.7 | 85 | 60.7 | 93 | 52.2 | 102 | 91.1 | 31 | 57.4 | 46 | 68.7 | 116 | 68.6 | 356 | 77.1 | 922 | 70.0 |
| 1 Watch | 43 | 35.5 | 8 | 53.3 | 66 | 47.1 | 101 | 56.7 | 11 | 9.8 | 24 | 44.4 | 38 | 56.7 | 86 | 50.9 | 227 | 49.1 | 604 | 45.8 |
| 2 Watch | 10 | 8.3 | 2 | 13.3 | 16 | 11.4 | 20 | 11.2 | 3 | 2.7 | 7 | 13.0 | 3 | 4.5 | 11 | 6.5 | 82 | 17.7 | 154 | 11.7 |
| 3 Watch | 3 | 2.5 | 1 | 6.7 | 3 | 2.1 | 1 | 0.6 | 0 | 0.0 | 0 | 0.0 | 0 | 0.0 | 3 | 1.8 | 24 | 5.2 | 35 | 2.7 |
| 4 Watch | 0 | 0.0 | 0 | 0.0 | 0 | 0.0 | 0 | 0.0 | 0 | 0.0 | 0 | 0.0 | 0 | 0.0 | 0 | 0.0 | 8 | 1.7 | 8 | 0.6 |
| 5 Watch | 0 | 0.0 | 0 | 0.0 | 0 | 0.0 | 0 | 0.0 | 0 | 0.0 | 1 | 1.9 | 0 | 0.0 | 0 | 0.0 | 2 | 0.4 | 3 | 0.2 |
| At least one Watch | 56 | 46.3 | 11 | 73.3 | 85 | 60.7 | 122 | 68.5 | 14 | 12.5 | 32 | 59.3 | 41 | 61.2 | 100 | 59.2 | 343 | 74.2 | 804 | 61.0 |
| 1 Reserve | 1 | 0.8 | 0 | 0.0 | 1 | 0.7 | 0 | 0.0 | 0 | 0.0 | 0 | 0.0 | 1 | 1.5 | 0 | 0.0 | 2 | 0.4 | 3 | 0.2 |
| 1 Unclassified** | 0 | 0.0 | 0 | 0.0 | 1 | 0.7 | 0 | 0.0 | 0 | 0.0 | 0 | 0.0 | 0 | 0.0 | 0 | 0.0 | 0 | 0.0 | 1 | 0.1 |
| *IV/IM: intravenous/intramuscular | | | | | | | | | | | | | | | | | | | | |
| **Ethambutol, Isoniazid and Pyrazinamide | | | | | | | | | | | | | | | | | | | | |

| **Supplementary Table 8**: **Total antibiotic use by country in ‘viral’ group (n; %)** | | | | | | | | | | | | | | | | | | | | | | |
| --- | --- | --- | --- | --- | --- | --- | --- | --- | --- | --- | --- | --- | --- | --- | --- | --- | --- | --- | --- | --- | --- | --- |
| Country (n) | Austria | | Germany | | Greece | | Latvia | | Slovenia | | Spain | | Switzerland | | Netherlands | | | United Kingdom | | | Total | |
|  | 46 | | 10 | | 107 | | 46 | | 24 | | 64 | | 8 | | 55 | | | 221 | | | 581 | |
| Received systemic antibiotics | 12 | 26.1 | 5 | 50.0 | 23 | 21.5 | 16 | 34.9 | 1 | 4.2 | 4 | 6.3 | 3 | 37.5 | 36 | 65.5 | 169 | | 76.5 | 269 | | 46.3 |
| **ALL % BELOW IS OUT OF THOSE WHO RECEIVED SYSTEMIC ANTIBIOTICS** | | | | | | | | | | | | | | | | | | | | | | |
| IV/IM* antibiotics | 10 | 83.3 | 3 | 60.0 | 20 | 87.0 | 9 | 56.3 | 1 | 100.0 | 2 | 50.0 | 3 | 100.0 | 31 | 86.1 | 155 | | 91.7 | 234 | | 87.0 |
| First Generation Cephalosporins | 1 | 8.3 | 0 | 0.0 | 0 | 0.0 | 0 | 0.0 | 0 | 0.0 | 1 | 25.0 | 0 | 0.0 | 0 | 0.0 | 2 | | 1.2 | 4 | | 1.5 |
| Second Generation Cephalosporins | 3 | 25.0 | 1 | 20.0 | 3 | 13.0 | 1 | 6.3 | 0 | 0.0 | 0 | 0.0 | 2 | 66.7 | 5 | 13.9 | 2 | | 1.2 | 17 | | 6.3 |
| Third Generation Cephalosporins | 3 | 25.0 | 0 | 0.0 | 9 | 39.1 | 6 | 37.5 | 0 | 0.0 | 2 | 50.0 | 1 | 33.3 | 21 | 58.3 | 121 | | 71.6 | 163 | | 60.6 |
| Fourth Generation Cephalosporins | 3 | 25.0 | 0 | 0.0 | 0 | 0.0 | 0 | 0.0 | 0 | 0.0 | 0 | 0.0 | 0 | 0.0 | 0 | 0.0 | 0 | | 0.0 | 3 | | 1.1 |
| Aminoglycoside | 0 | 0.0 | 0 | 0.0 | 2 | 8.7 | 1 | 6.3 | 0 | 0.0 | 0 | 0.0 | 0 | 0.0 | 5 | 13.9 | 13 | | 7.7 | 21 | | 7.8 |
| Carbapenems | 0 | 0.0 | 0 | 0.0 | 0 | 0.0 | 0 | 0.0 | 0 | 0.0 | 0 | 0.0 | 0 | 0.0 | 1 | 2.8 | 3 | | 1.8 | 4 | | 1.5 |
| DHFR inhibitors | 0 | 0.0 | 1 | 20.0 | 0 | 0.0 | 1 | 6.3 | 0 | 0.0 | 0 | 0.0 | 0 | 0.0 | 1 | 2.8 | 3 | | 1.8 | 6 | | 2.2 |
| Fluoroquinolones | 0 | 0.0 | 0 | 0.0 | 0 | 0.0 | 0 | 0.0 | 0 | 0.0 | 0 | 0.0 | 0 | 0.0 | 1 | 2.8 | 4 | | 2.4 | 5 | | 1.9 |
| Glycopeptides | 0 | 0.0 | 0 | 0.0 | 0 | 0.0 | 0 | 0.0 | 0 | 0.0 | 1 | 25.0 | 0 | 0.0 | 2 | 5.6 | 7 | | 4.1 | 10 | | 3.7 |
| Imidazoles | 1 | 8.3 | 0 | 0.0 | 0 | 0.0 | 0 | 0.0 | 0 | 0.0 | 0 | 0.0 | 0 | 0.0 | 1 | 2.8 | 2 | | 1.2 | 4 | | 1.5 |
| Lincosamides | 1 | 8.3 | 0 | 0.0 | 1 | 4.3 | 0 | 0.0 | 0 | 0.0 | 0 | 0.0 | 0 | 0.0 | 1 | 2.8 | 2 | | 1.2 | 5 | | 1.9 |
| Macrolides | 0 | 0.0 | 1 | 20.0 | 1 | 4.3 | 2 | 12.5 | 0 | 0.0 | 0 | 0.0 | 0 | 0.0 | 2 | 5.6 | 43 | | 25.4 | 49 | | 18.2 |
| Nitrofurantoin | 0 | 0.0 | 0 | 0.0 | 0 | 0.0 | 0 | 0.0 | 0 | 0.0 | 0 | 0.0 | 0 | 0.0 | 0 | 0.0 | 0 | | 0.0 | 0 | | 0.0 |
| Oxazolidinones | 0 | 0.0 | 0 | 0.0 | 0 | 0.0 | 0 | 0.0 | 0 | 0.0 | 0 | 0.0 | 0 | 0.0 | 0 | 0.0 | 0 | | 0.0 | 0 | | 0.0 |
| Penicillin/Beta-lactamase Inhibitor Combinations | 2 | 16.7 | 1 | 20.0 | 1 | 4.3 | 1 | 6.3 | 1 | 100.0 | 0 | 0.0 | 0 | 0.0 | 7 | 19.4 | 53 | | 31.4 | 66 | | 24.5 |
| Penicillins | 0 | 0.0 | 2 | 40.0 | 9 | 39.1 | 7 | 43.8 | 0 | 0.0 | 2 | 50.0 | 0 | 0.0 | 10 | 27.8 | 33 | | 19.5 | 63 | | 23.4 |
| Rifamycins | 0 | 0.0 | 0 | 0.0 | 0 | 0.0 | 0 | 0.0 | 0 | 0.0 | 0 | 0.0 | 0 | 0.0 | 0 | 0.0 | 0 | | 0.0 | 0 | | 0.0 |
| Tetracyclines | 0 | 0.0 | 0 | 0.0 | 0 | 0.0 | 0 | 0.0 | 0 | 0.0 | 0 | 0.0 | 0 | 0.0 | 0 | 0.0 | 0 | | 0.0 | 0 | | 0.0 |
| Other** | 0 | 0.0 | 0 | 0.0 | 0 | 0.0 | 0 | 0.0 | 0 | 0.0 | 0 | 0.0 | 0 | 0.0 | 1 | 2.8 | 1 | | 0.6 | 2 | | 0.7 |
| Unknown | 0 | 0.0 | 0 | 0.0 | 0 | 0.0 | 0 | 0.0 | 0 | 0.0 | 0 | 0.0 | 0 | 0.0 | 0 | 0.0 | 0 | | 0.0 | 0 | | 0.0 |
| 1 Access | 5 | 41.7 | 4 | 80.0 | 11 | 47.8 | 10 | 62.5 | 1 | 100.0 | 1 | 25.0 | 0 | 0.0 | 18 | 50.0 | 71 | | 42.0 | 120 | | 44.6 |
| 2 Access | 0 | 0.0 | 0 | 0.0 | 1 | 4.3 | 0 | 0.0 | 0 | 0.0 | 1 | 25.0 | 0 | 0.0 | 3 | 8.3 | 7 | | 4.1 | 12 | | 4.5 |
| 6 Access | 0 | 0.0 | 0 | 0.0 | 0 | 0.0 | 0 | 0.0 | 0 | 0.0 | 0 | 0.0 | 0 | 0.0 | 0 | 0.0 | 1 | | 0.6 | 1 | | 0.4 |
| At least one Access | 5 | 41.7 | 4 | 80.0 | 12 | 52.2 | 10 | 62.5 | 1 | 100.0 | 2 | 50.0 | 0 | 0.0 | 21 | 58.3 | 80 | | 47.3 | 135 | | 50.2 |
| 1 Watch | 9 | 75.0 | 2 | 40.0 | 13 | 56.5 | 9 | 56.3 | 0 | 0.0 | 0 | 0.0 | 3 | 100.0 | 18 | 50.0 | 93 | | 55.0 | 147 | | 54.6 |
| 2 Watch | 0 | 0.0 | 0 | 0.0 | 0 | 0.0 | 0 | 0.0 | 0 | 0.0 | 2 | 50.0 | 0 | 0.0 | 6 | 16.7 | 43 | | 25.4 | 51 | | 19.0 |
| 3 Watch | 0 | 0.0 | 0 | 0.0 | 0 | 0.0 | 0 | 0.0 | 0 | 0.0 | 0 | 0.0 | 0 | 0.0 | 0 | 0.0 | 14 | | 8.3 | 14 | | 5.2 |
| 4 Watch | 0 | 0.0 | 0 | 0.0 | 0 | 0.0 | 0 | 0.0 | 0 | 0.0 | 0 | 0.0 | 0 | 0.0 | 1 | 2.8 | 0 | | 0.0 | 1 | | 0.4 |
| 5 Watch | 0 | 0.0 | 0 | 0.0 | 0 | 0.0 | 0 | 0.0 | 0 | 0.0 | 0 | 0.0 | 0 | 0.0 | 0 | 0.0 | 1 | | 0.6 | 1 | | 0.4 |
| At least one Watch | 9 | 75.0 | 2 | 40.0 | 13 | 56.5 | 9 | 56.3 | 0 | 0.0 | 2 | 50.0 | 3 | 100.0 | 25 | 69.4 | 153 | | 90.5 | 216 | | 80.3 |
| 1 Reserve | 0 | 0.0 | 0 | 0.0 | 0 | 0.0 | 0 | 0.0 | 0 | 0.0 | 0 | 0.0 | 0 | 0.0 | 0 | 0.0 | 0 | | 0.0 | 0 | | 0.0 |
| *IV/IM: intravenous/intramuscular | | | | | | | | | | | | | | | | | | | | | | |
| **Ethambutol, Isoniazid and Pyrazinamide | | | | | | | | | | | | | | | | | | | | | | |

| **Supplementary Table 9: Age-based comparison of patients with an initial viral or non-infectious working diagnosis (n=251) receiving / not receiving empiric antibiotics** | | | |
| --- | --- | --- | --- |
|  | < 5 years | ≥5 years | p-value* |
| initial presumed viral/ non-infectious etiology receiving antibiotics (96/252; 38.0%) | 71 (45.5%) | 24 (25.3%) | <0.01 |
| initial presumed viral/ non-infectious etiology not receiving antibiotics (156/252; 62.0%) | 85 (54.5%) | 71 (74.7%) |  |
| *chi-square |  |  |  |

| **Supplementary Table 10: The most common bacterial pathogens in the ‘bacterial’ group and the most common viral pathogens in the ‘viral’ group** | | | | | | |
| --- | --- | --- | --- | --- | --- | --- |
| **Bacterial pathogens** | number of patients | | % of bacterial patients | **Viral pathogens** | number of patients | % of viral patients |
| Escherichia coli (E. Coli) | 213 | | 23.5 | Influenza A/B | 135 | 20.5 |
| Streptococcus Group A (Strep. pyogenes) | 125 | | 13.8 | Rhinovirus/Enterovirus | 127 | 19.3 |
| Staphylococcus aureus | 121 | | 13.3 | Respiratory syncytial virus (RSV) | 85 | 12.9 |
| Streptococcus pneumoniae (pneumococcus) | 55 | | 6.1 | Adenovirus | 76 | 11.6 |
| Pseudomonas aeruginosa | 33 | | 3.6 | Epstein-Barr Virus (EBV) | 45 | 6.8 |
| Neisseria meningitidis (meningococcus) | 30 | | 3.3 | Measles | 30 | 4.6 |
| Mycoplasma pneumoniae | 29 | | 3.2 | Rotavirus | 27 | 4.1 |
| Campylobacter spp. | 28 | | 3.1 | Metapneumovirus | 16 | 2.4 |
| Salmonella spp. | 26 | | 2.9 | Herpes simplex type 1 | 14 | 2.1 |
| Staphylococcus - coagulase negative (includes capitis, epidermidis, haemolyticus, hominis) | 22 | | 2.4 | Varicella zoster virus (VZV) | 14 | 2.1 |
| Enterobacter cloacae | 14 | | 1.5 | Coronavirus | 12 | 1.8 |
| Haemophilus influenzae* | 14 | | 1.5 | Norovirus | 12 | 1.8 |
| Enterococcus faecalis | 13 | | 1.4 | Parainfluenza type 3 | 10 | 1.5 |
| Borrelia burdorferi | 11 | | 1.2 | Parvovirus | 8 | 1.2 |
| Klebsiella pneumoniae | 11 | | 1.2 | Tick borne encephalitis virus | 7 | 1.1 |
| Streptococcus viridans group (includes mitis, mutans, salivarius, sanguinis) | 11 | | 1.2 | Hepatitis A virus | 6 | 0.9 |
| Bacteroides fragilis | 8 | | 0.9 | Bocavirus | 5 | 0.8 |
| Kingella kingae | 7 | | 0.8 | Cytomegalovirus (CMV) | 5 | 0.8 |
| Mycobacterium tuberculosis | 7 | | 0.8 | Human herpesvirus 6 (HHV6) | 4 | 0.6 |
| Streptococcus - alpha haemolytic, no further information | 7 | | 0.8 | Herpes simplex* | 3 | 0.5 |
| Bordetella pertussis | 6 | | 0.7 | Parainfluenza type 4 | 3 | 0.5 |
| Streptococcus Group F (includes anginosus, milleri) | 6 | | 0.7 | Dengue virus | 2 | 0.3 |
| Clostridium difficile | 5 | | 0.6 | human herpes type 7 | 2 | 0.3 |
| Klebsiella oxytoca | 5 | | 0.6 | Parainfluenza type 2 | 2 | 0.3 |
| Proteus spp. | 5 | | 0.6 | Picornavirus | 2 | 0.3 |
| Streptococcus Group B (includes Strep.agalactae) | 5 | | 0.6 | Parechovirus | 2 | 0.3 |
| Citrobacter freudii | 4 | | 0.4 | HIV 1 | 1 | 0.2 |
| Enterococcus spp. | 4 | | 0.4 | Parainfluenza type 1 | 1 | 0.2 |
| Fusobacterium spp. | 4 | | 0.4 | Parainfluenza type 5 | 1 | 0.2 |
| Anaerobes* | 4 | | 0.4 |  |  |  |
| Mycoplasma spp. | 4 | | 0.4 |  |  |  |
| Shigella spp. | 4 | | 0.4 |  |  |  |
| Yersinia enterocolitica | 4 | | 0.4 |  |  |  |
| Acinetobacter spp. | 3 | | 0.3 |  |  |  |
| Clostridium perfringens | 3 | | 0.3 |  |  |  |
| Enterococcus faecium | 3 | | 0.3 |  |  |  |
| Coliforms | 3 | | 0.3 |  |  |  |
| Streptococcus Group C | 3 | | 0.3 |  |  |  |
| Unidentified bacteria | 3 | | 0.3 |  |  |  |
| Aerococcus urinae | 2 | | 0.2 |  |  |  |
| Citrobacter spp. | 2 | | 0.2 |  |  |  |
| Corynebacteria spp. | 2 | | 0.2 |  |  |  |
| Klebsiella spp. | 2 | | 0.2 |  |  |  |
| Moraxella catarrhalis | 2 | | 0.2 |  |  |  |
| Neisseria spp. | 2 | | 0.2 |  |  |  |
| Prevotella spp. | 2 | | 0.2 |  |  |  |
| Serratia marcescens | 2 | | 0.2 |  |  |  |
| Stenotrophomonas maltophilia | 2 | | 0.2 |  |  |  |
| Streptococcus anginosus | 2 | | 0.2 |  |  |  |
| acinetobacter calcoaceticus-baumannii Komplex | 1 | | 0.1 |  |  |  |
| Actinobaculum spp. | 1 | | 0.1 |  |  |  |
| Actinomyces odontolyticus | 1 | | 0.1 |  |  |  |
| Bacteroides thetaiotaomicron | 1 | | 0.1 |  |  |  |
| bartonella henselae | 1 | | 0.1 |  |  |  |
| Borellia spp. | 1 | | 0.1 |  |  |  |
| Chlamydia pneumoniae | 1 | | 0.1 |  |  |  |
| Chlamydia spp. | 1 | | 0.1 |  |  |  |
| Eikenella corrodens | 1 | | 0.1 |  |  |  |
| Erlichia spp. | 1 | | 0.1 |  |  |  |
| Francisella tularensis | 1 | | 0.1 |  |  |  |
| Gordonia spp. | 1 | | 0.1 |  |  |  |
| Granulicatella elegans | 1 | | 0.1 |  |  |  |
| Haemophilus parainfluenzae | 1 | | 0.1 |  |  |  |
| Hafnia alvei | 1 | | 0.1 |  |  |  |
| Lactobacillus rhamnosus | 1 | | 0.1 |  |  |  |
| Micrococcus spp. | 1 | | 0.1 |  |  |  |
| Morganella morganii | 1 | | 0.1 |  |  |  |
| Mycobacterium spp. | 1 | | 0.1 |  |  |  |
| Pseudomonas spp. | 1 | | 0.1 |  |  |  |
| Roseomonas mucosa | 1 | | 0.1 |  |  |  |
| Staphylococcus warneri | 1 | | 0.1 |  |  |  |
| streptococcus constellatus | 1 | | 0.1 |  |  |  |
| Streptococcus Group G (includes Strep.dysgalactiae) | 1 | | 0.1 |  |  |  |
| * unspecified | |  |  |  |  |  |

| **Supplementary Table 11: systemic antibiotic use in three most common bacterial pathogens (*Escherichia coli*, *Streptococcus pyogenes* (GAS) and *Staphylococcus aureus* in the ‘bacterial’ group– where single pathogen isolated (n; %)** | | | | | | |
| --- | --- | --- | --- | --- | --- | --- |
|  | patients with  only *Escherichia coli* (n=188) | | patients with  only GAS  (n=115) | | patients with only *Staphylococcus aureus*  (n=104) | |
| First Generation Cephalosporins | 14 | 7.4 | 4 | 3.5 | 4 | 3.8 |
| Second Generation Cephalosporins | 36 | 19.1 | 21 | 18.3 | 9 | 8.7 |
| Third Generation Cephalosporins | 64 | 34.0 | 35 | 30.4 | 35 | 33.7 |
| Fourth Generation Cephalosporins | 2 | 1.1 | 0 | 0.0 | 1 | 1.0 |
| Aminoglycoside | 44 | 23.4 | 6 | 5.2 | 9 | 8.7 |
| Carbapenems | 10 | 5.3 | 3 | 2.6 | 1 | 1.0 |
| DHFR inhibitors | 11 | 5.9 | 0 | 0.0 | 1 | 1.0 |
| Fluoroquinolones | 8 | 4.3 | 2 | 1.7 | 3 | 2.9 |
| Glycopeptides | 3 | 1.6 | 4 | 3.5 | 11 | 10.6 |
| Imidazoles | 7 | 3.7 | 3 | 2.6 | 7 | 6.7 |
| Lincosamides | 1 | 0.5 | 20 | 17.4 | 31 | 29.8 |
| Macrolides | 5 | 2.7 | 8 | 7.0 | 3 | 2.9 |
| Nitrofurantoin | 0 | 0.0 | 0 | 0.0 | 0 | 0.0 |
| Oxazolidinones | 0 | 0.0 | 1 | 0.9 | 1 | 1.0 |
| Penicillin/Beta-lactamase Inhibitor Combinations | 42 | 22.3 | 24 | 20.9 | 27 | 26.0 |
| Penicillins | 20 | 10.6 | 44 | 38.3 | 41 | 39.4 |
| Rifamycins | 0 | 0.0 | 0 | 0.0 | 0 | 0.0 |
| Tetracyclines | 0 | 0.0 | 0 | 0.0 | 0 | 0.0 |
| Other | 1 | 0.5 | 0 | 0.0 | 6 | 5.8 |
| Unknown | 0 | 0.0 | 0 | 0.0 | 1 | 1.0 |
| At least one Access | 97 | 51.6 | 83 | 72.2 | 86 | 82.7 |
| At least one Watch | 120 | 63.3 | 55 | 47.8 | 51 | 49.0 |

| **Supplementary Table 12: antibiotic use in three most common viral pathogens (Influenza A and B, Rhino-/Enterovirus and respiratory syncytial virus (RSV)) (n; %)** | | | | | | |
| --- | --- | --- | --- | --- | --- | --- |
|  | patients with only Influenza A and B (n=119) | | patients with only Rhino-/Enterovirus (n=89) | | patients with only  RSV (n=66) | |
|  |  |  |  |  |  |  |
| Received systemic antibiotics | 42 | 35.3 | 57 | 64.0 | 44 | 66.7 |
| **ALL % BELOW IS OUT OF THOSE WHO RECEIVED SYSTEMIC ANTIBIOTICS** | | | | | | |
| IV/IM antibiotics* | 38 | 90.5 | 52 | 91.2 | 35 | 83.3 |
| First Generation Cephalosporins | 0 | 0.0 | 0 | 0.0 | 0 | 0.0 |
| Second Generation Cephalosporins | 3 | 7.1 | 1 | 1.8 | 1 | 2.4 |
| Third Generation Cephalosporins | 24 | 57.1 | 42 | 73.7 | 24 | 57.1 |
| Fourth Generation Cephalosporins | 0 | 0.0 | 1 | 1.8 | 0 | 0.0 |
| Aminoglycoside | 3 | 7.1 | 6 | 10.5 | 4 | 9.5 |
| Carbapenems | 1 | 2.4 | 0 | 0.0 | 1 | 2.4 |
| DHFR inhibitors | 2 | 4.8 | 0 | 0.0 | 1 | 2.4 |
| Fluoroquinolones | 3 | 7.1 | 1 | 1.8 | 0 | 0.0 |
| Glycopeptides | 1 | 2.4 | 3 | 5.3 | 2 | 4.8 |
| Imidazoles | 2 | 4.8 | 1 | 1.8 | 0 | 0.0 |
| Lincosamides | 1 | 2.4 | 0 | 0.0 | 0 | 0.0 |
| Macrolides | 8 | 19.0 | 5 | 8.8 | 15 | 35.7 |
| Nitrofurantoin | 0 | 0.0 | 0 | 0.0 | 0 | 0.0 |
| Oxazolidinones | 0 | 0.0 | 0 | 0.0 | 0 | 0.0 |
| Penicillin/Beta-lactamase Inhibitor Combinations | 17 | 40.5 | 7 | 12.3 | 12 | 28.6 |
| Penicillins | 4 | 9.5 | 20 | 35.1 | 11 | 26.2 |
| Rifamycins | 0 | 0.0 | 0 | 0.0 | 0 | 0.0 |
| Tetracyclines | 0 | 0.0 | 0 | 0.0 | 0 | 0.0 |
| Other | 0 | 0.0 | 0 | 0.0 | 1 | 2.4 |
| Unknown | 0 | 0.0 | 0 | 0.0 | 0 | 0.0 |
| At least one Access | 25 | 59.5 | 28 | 49.1 | 21 | 50.0 |
| At least one Watch | 31 | 73.8 | 48 | 84.2 | 34 | 81.0 |
| *IV/IM: intravenous/intramuscular | |  |  |  |  |  |

Supplementary Figure 1**: PERFORM phenotyping algorithm**

Supplementary Figure 2**: Consistency of initial diagnosis with final diagnosis and antibiotic prescription**

LRTI: lower respiratory tract infection

URTI/ENT: upper respiratory tract infection, ear nose throat

MUSCULOSKELETAL: musculoskeletal infection

CNS: central nervous system infection

GI: gastrointestinal infection

SURG/INTRA-ABDO: surgical /intra-abdominal infection

SOFT TISSUE: soft tissue infection

UTI: urinary tract infection
